# Dose- and time-dependent manners of moxifloxacin induced liver injury by targeted metabolomics study

**DOI:** 10.3389/fphar.2022.994821

**Published:** 2022-09-16

**Authors:** Ting Hu, Yuan Sun, Zhuoling An

**Affiliations:** Beijing Chao-Yang Hospital, Capital Medical University, Beijing, China

**Keywords:** moxifloxacin, liver injury, metabolomics, fatty acid, fatty acyl carnitines, dehydroepiandrosterone

## Abstract

Moxifloxacin is the most widely prescribed antibiotics due to its excellent oral bioavailability and broad-spectrum antibacterial effect. Despite of its popularity, the rare and severe liver injury induced by moxifloxacin is a big concern that cannot be ignored in clinical practice. However, the early warning and related metabolic disturbances of moxifloxacin induced hepatoxicity were rarely reported. In this study, the dose- and time-dependent manners of moxifloxacin induced liver injury were investigated by a targeted metabolomics method. In dose-dependent experiment, three different dosages of moxifloxacin were administered to the rats, including 36 mg kg^−1^ d^−1^, 72 mg kg^−1^ d^−1^, and 108 mg kg^−1^ d^−1^. In time-dependent experiment, moxifloxacin was orally administered to the rats for 3, 7 or 14 consecutive days. Pathological analysis showed that moxifloxacin caused obvious transient hepatotoxicity, with the most serious liver injury occurred in the 7 days continuous administration group. The transient liver injury can be automatically restored over time. Serum levels of liver function related biochemical indicators, including ALT, AST, TBIL, alkaline phosphatase, superoxide dismutase, and malondialdehyde, were also measured for the evaluation of liver injury. However, these indicators can hardly be used for the early warning of hepatotoxicity caused by moxifloxacin due to their limited sensitivity and significant hysteresis. Targeted metabolomics study demonstrated that serum concentrations of fatty acyl carnitines, fatty acids and dehydroepiandrosterone can change dynamically with the severity of moxifloxacin related liver injury. The elevated serum levels of fatty acyl carnitine, fatty acid and dehydroepiandrosterone were promising in predicting the hepatotoxicity induced by moxifloxacin.

## Introduction

Moxifloxacin is a fourth-generation fluoroquinolone antibiotic approved for use in the United States in 1999 (Soto et al., 2002). It is on the World Health Organization’s List of Essential Medicines and is widely used to treat bacterial infections, such as pneumonia, conjunctivitis, endocarditis, *tuberculosis*, and sinusitis (Balfour and Wiseman, 1999; Xu et al., 2017). Moxifloxacin is a broad-spectrum antibiotic that is active against both Gram-negative and Gram-positive bacteria. Like other fluoroquinolone anti-bacterial agents, moxifloxacin inhibits growth of susceptible bacteria by inhibiting bacterial DNA topoisomerases (Balfour and Wiseman, 1999; Xu et al., 2017). Despite of the good and broad-spectrum antibacterial effect, post-marketing clinical observations have demonstrated that moxifloxacin can cause severe hepatotoxicity with drug-induced hepatitis, abnormal liver function and jaundice (Chalasani et al., 2015; Xu et al., 2017). The European Medicines Agency recommends the use of the oral form of moxifloxacin be restricted to infections that cannot be treated with other antimicrobials or for which other antibiotics have failed (Paterson et al., 2012). The rare but severe hepatotoxicity induced by moxifloxacin is the main obstacle for its wide-spread application in patients. Due to the few treatments for mitigating idiosyncratic acute poisoning, moxifloxacin related hepatotoxicity carries significant mortality (Paterson et al., 2012; Chalasani et al., 2015).

The predominant features of moxifloxacin induced hepatotoxicity were the short latency and abrupt onset of injury (Orman et al., 2011; Paterson et al., 2012). As reported by Eric and coworkers, the median time to onset of drug-induced liver injury (DILI) was 4 days from starting the medication of fluoroquinolones (Orman et al., 2011). And median time to onset of abnormal liver function tests was 8.5 days (Orman et al., 2011). The hepatotoxicity of moxifloxacin was manifested histologically as intrahepatic cholestasis, portal and lobular inflammation, and increased number of plasma cells in the infiltrate (Soto et al., 2002; Orman et al., 2011). When severe liver injury occurs, most of the hepatocyte parenchyma was replaced by a ductular reaction embedded in dense fibrosis by the time of liver transplant (Soto et al., 2002). The short latency, frequent immunoallergic features, heightened injury that has been described upon re-exposure, and the lack of a common pattern of metabolism of the various fluoroquinolones argue for a hypersensitivity reaction (Schmid et al., 2006). Nevertheless, the pathophysiology of moxifloxacin related hepatotoxicity still need to be elucidated. Significant questions remain in understanding the exact molecular mechanisms and metabolic pathways involved in the development and progression of moxifloxacin induced hepatotoxicity.

Clinically, the diagnosis of DILI is based on serum biochemical indicators related to liver function. However, abnormal increases of these indicators, including changes in alanine aminotransferase (ALT), aspartate aminotransferase (AST), and alkaline phosphatase (ALP), are secondary to appear after hepatocyte injury (Katarey and Verma, 2016; Chalasani et al., 2021). These traditional serum indicators can hardly be used for the early warning of severe hepatotoxicity caused by moxifloxacin. Targeted metabolomics is defined as the quantification of specific small-molecule metabolites on specific metabolic pathway of the organism (Johnson et al., 2016; Jacob et al., 2019). It has been emerged as a useful tool for identifying potential biomarkers and clarifying the mechanisms associated with diseases, drug effects and toxicity (Johnson et al., 2016). Targeted metabolomics has been widely used for the early warning and mechanism research of liver injury induced by acetaminophen, anti-tuberculosis drugs, triptolide, and doxorubicin (Combrink et al., 2020; Zhao et al., 2020; Yao et al., 2021). The disturbance of bile acids, glutathione, lipid and purine were the most frequently reported metabolic pathways related to DILI (Mattes et al., 2014; Liu et al., 2020). Although targeted metabolomics has been proved to play an important role in the early diagnosis and pathogenesis clarification of DILI, it has not yet been used to investigate the dose- and time-dependent manner of moxifloxacin induced liver injury.

In this study, a targeted metabolomics method was applied to explore the disturbance of endogenous small molecules and their metabolic pathways after moxifloxacin administration in rats. Targeted metabolomics was conducted by using ultra-high performance liquid chromatography coupled with tandem mass spectrometry (UHPLC-MS/MS). Dose- and time-dependent hepatotoxicity induced by moxifloxacin were comprehensively investigated to identify potential biomarkers that may aid in the early prediction of moxifloxacin induced liver injury.

## Materials and methods

### Reagents and materials

Acetonitrile, methanol and isopropyl alcohol of mass spectrometry (MS) grade were purchased from Fisher Scientific (Pittsburgh, PA, United States). MS grade formic acid was obtained from Sigma-Aldrich (St. Louis, MO, United States). Ultrapure water was prepared by a Milli-Q purification system (Bedford, MA, United States). Unlabeled chemical standards were obtained from Cayman Chemical (Ann Arbor, MI, United States), Sigma-Aldrich, Bidepharm (Shanghai, China) or Steraloids (Newport, RI, United States). Stable isotope labeled internal standards (ISs) were purchased from Cambridge Isotope Laboratories (Cambridge, MA, United States), Cayman Chemical or Steraloids. Moxifloxacin was the product of Bayer Schering Pharma AG (Leverkusen, Germany).

### Animal experiments

All the animal experiments were conducted in accordance with institutional guidelines and ethics approved by the Animal Ethics Committee of Beijing Chao-Yang Hospital affiliated with Beijing Capital Medical University. Male Wistar rats aged 8 weeks (body weights: 200 ± 5 g) were purchased from Beijing Vital River Laboratory Animal Technology Co., Ltd. (Beijing. China). All the rats were kept in animal house of specific pathogen free grade at 23 ± 3°C and 50% ± 10% relative humidity with a 12 h light and dark cycle. The rats had free access to a normal chow diet and tap water.

After 2 weeks adaptive feeding, the rats were randomly divided into control and different dosing groups, with 6 animals in each group. Moxifloxacin was prepared with normal saline. In moxifloxacin dose-dependent experiment, the rats were administered with oral dosages of 36 mg kg^−1^ d^−1^ (Low dosage, LD), 72 mg kg^−1^ d^−1^ (Medium dosage, MD), or 108 mg kg^−1^ d^−1^ (High dosage, HD). Low dosage of moxifloxacin (36 mg kg^−1^ d^−1^) was calculated by converting the human’s dosage using a recognized coefficient of 6.3. Medium dosage and high dosage were twice and three times of the low dosage, respectively. In time-dependent experiment, moxifloxacin of different dosages was orally administered to the rats for 3, 7 or 14 consecutive days. While the same dosages of saline were given to animals in control groups. After receiving the last dose of moxifloxacin, the rats were fasted overnight and then euthanized by exposure to isoflurane. Specifically, rats were exposed to 5% isoflurane carried in oxygen at a fill rat of 20% cv/min until loss of righting reflex, followed by stopping isoflurane administration and switching to 100% CO_2_ (30% cv/min). Serum samples were isolated from blood and used for biochemical index detection and targeted metabolomics analysis. Liver tissue was immediately harvested after blood collection and soaked in fixative solution. The serum samples were stored at −80°C until targeted metabolomics detection.

### Biochemical and histopathological analysis

Serum biochemical indicators, including AST, ALT, total bilirubin (TBIL), ALP, superoxide dismutase (SOD), and malondialdehyde (MDA) were measured by using a clinical biochemical analyzer (AU400, Olympus, Japan). Liver tissue was harvested and soaked in 10% buffered formalin phosphate solution for histological sectioning. The formalin-fixed, paraffin-embedded liver tissue samples were ultrasectioned (4–5 μm thickness), stained with hematoxylin and eosin (H&E) and examined under a light microscope.

### Biological sample processing for targeted metabolomics

ISs solution was prepared by mixing thymine-*d4*, valine-*d8*, phenylalanine-*d8*, 17-hydroxyprogesterone-*d8*, cholic acid-*d4*, chenodeoxycholic acid-*d4* and glycocholic acid-*d4* at a concentration level of 400 ng/ml for each isotopic compound. For biological sample pretreatment, each 50 μl rat serum was mixed with 10 μl ISs solution and 140 μl cold methanol (−20°C). The mixture was vortexed for 5 min. The resulting mixture was applied for centrifuging at 13800 g for 10 min. The supernatants were collected and used for targeted metabolomics analysis.

### Targeted metabolomics analysis by UHPLC-MS/MS

A targeted metabolomics method previously established in our laboratory were employed for metabolites quantification in serum samples of the rats (Hu et al., 2020). A Spark Holland liquid chromatography system (Spark, Holland) coupled with an API 5500 mass spectrometer (AB Sciex, Canada) with an electrospray ionization (ESI) source was used for metabolomics detection. A total of 289 metabolites, can be quantified with this UHPLC-MS/MS based targeted metabolomics method, including acyl carnitines, amino acids and derivatives, nucleotides and derivatives, bile acids, fatty acids, sugar and derivatives, organic acids and derivatives, steroids, bioamines and derivatives, vitamins and derivatives, pteridines and derivatives. Quantification curves were prepared from the standard mixture at 12 different concentrations (0.2, 0.5, 2, 5, 20, 50, 100, 200, 500, 1,000, 2000, 5,000 ng/ml) using methanol as the dilution solvent. Each 50 μl standard mixture was spiked with 10 μl IS mixture solution, 50 μl water and 90 μl methanol to get the standard curve sample.

A Waters HSS T3 (150 × 2.1 mm, 3.5 μm) was used for chromatographic separation with column temperature maintained at 20°C. The injection volume was 5 μl. Mobile phase A was water with 0.1% formic acid and mobile phase B was acetonitrile/isopropyl alcohol 7:2 (v/v). The flow rate of the mobile phase was set to 0.5 ml/min. Gradient elution was used as follows: 0 min, 1% B; 4 min, 10% B; 8 min, 50% B; 15 min, 80% B; 25 min, 100% B; 27 min, 100% B. The elution condition was returned to the initial state over a period of 2 min.

All metabolites and isotope ISs were analyzed in a single-injection using both negative and positive modes with rapid polarity switching (50 ms) and advanced s-MRM algorithm. The MS source parameters were set as follows: electrospray voltage at −4500 V for negative mode and 5500 V at positive mode, source temperature at 600°C, GS1 at 60 psi, GS2 at 60 psi, curtain gas at 40 psi. For MRM parameter optimization, each metabolite of 1 μg/ml concentration was injected to MS by a syringe pump at a flow rate of 10 μl/min, and the MRM ion pairs, collision energies (CEs), declustering potentials (DPs) and other parameters were optimized automatically (Supplementary Table S1). For more details on the methodology, please refer to the previously published study by the authors (Hu et al., 2020).

All the metabolites listed in Supplementary Table S1 have standards and can be classified as the category of identified compounds according to a previously report (Sumner et al., 2007). Besides, some of the fatty acyl carnitines without standards were identified by the high-resolution MS and MS/MS data. These fatty acyl carnitines can be classified as the category of putatively annotated compounds.

### Data processing and statistical analysis

UHPLC-MS/MS data was processed by using MultiQuant software (version 3.0.2, AB SCIEX). Peak area of each analyte was calibrated by its corresponding IS peak area. The resulting peak area ratios of the standards were plotted against the real concentrations to construction calibration curves by the least-square method with a *1/x* ^*2*^ weighting factor. The relative concentration of each metabolite was calculated according to the peak area ratio of an analyte to the corresponding IS. The absolute concentration of each metabolite was calculated according to the calibration curve. SIMCA 14.1 (Umetrics AB, Umeå, Sweden) was employed for principal component analysis (PCA) and partial least-squares discriminant analysis (PLS-DA). Prism 7.0 (GraphPad Software, San Diego, CA) was employed for bilateral *t*-test, one-way analysis of variance (ANOVA) analysis and receiver operating characteristic curves (ROC) analysis. IBM SPSS 21 (Armonk, New York, United States) was used for Spearman’s rank correlation analysis. *p* value less than 0.05 was considered as the level of statistical significance. Heat map and metabolic pathway enrichment analysis were performed in an open source tool of MetaboAnalyst 5.0 (https://www.metaboanalyst.ca/). Circos plot was generated also generated in an open online tool (http://mkweb.bcgsc.ca/tableviewer/).

## Results

### Histopathological analysis and clinical biochemical indicators

**FIGURE 1:**
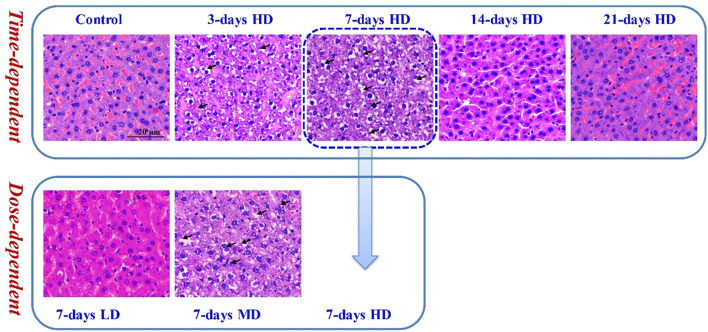
Representative pathological images of H&E staining under light microscopy. Significant hepatocellular ballooning, nuclear pyknosis and karyorrhexis were observed in the H&E staining liver tissue of the 3-day HD group, 7-day MD group and 7-day HD group.

To investigate the liver injury caused by moxifloxacin administration, H&E staining was used for pathological examination of rat liver tissues. As shown in Figure 1, continuous administration of moxifloxacin for 3- or 7-day resulted in extensive liver damage. Significant hepatocellular ballooning, disordered arrangement of liver lobule structure and markedly elevated cellular apoptosis were observed in the liver tissue harvested from the 3- and 7- day administration groups. Nuclear pyknosis, karyorrhexis, inflammatory infiltration and focal necrosis were also observed in the same groups. For groups with different dosages in the 7 consecutive days administration animals, the degree of liver injury was positively correlated with moxifloxacin dosages (Lower panel of Figure 1). Histological analysis indicated that the HD groups showed more severe liver damage than the MD and LD groups in the 7-day administration animals. However, the liver injury of the 14- and 21-day administration groups was not obvious. Only mild liver injury was observed on pathological section of the 14-day administration groups. In the 21-day administration groups, almost no liver injury was observed from the pathological sections.

Serum levels of biochemical indicators, including ALT, AST, TBIL, ALP, SOD, and MDA, were also measured to evaluate the hepatotoxicity of moxifloxacin (Table 1). Compared with the control group, moxifloxacin treatment animals showed increased levels of serum ALT and AST. The most significant changes of ALT occurred in the 3-day administration groups. Though the levels of ALT and AST were significantly difference between control and dosing groups, their fold changes in different dosing groups were less than 2. Changes in TBIL levels appeared to be related to the time of moxifloxacin administration, and the most significant increase of TBIL occurred in the 14- and 21-day administration groups. A downward trend of serum ALP was observed in most of the dosing groups compared to the control group, which was contrary to expectations. Indicators related to peroxidation, including SOD and MDA, were generally not disturbed by moxifloxacin administration.

**TABLE 1:** Levels of biochemical indicators in serum.

Time	Dose	ALT (U/L)	AST (U/L)	TBIL (umol/L)	ALP (U/L)	SOD (U/mgprot)	MDA (nmol/mgprot)
0 day	0 mg/kg	35.2 ± 7.9	107.7 ± 11.3	0.38 ± 0.21	191.7 ± 22.9	275.5 ± 32.5	4.40 ± 0.37
3-day	36 mg/kg	53.3 ± 9.8^**^	146.0 ± 34.9^*^	0.42 ± 0.28	174.5 ± 32.9	242.1 ± 14.4	4.39 ± 0.64
	72 mg/kg	63.7 ± 11.7^**^	161.5 ± 34.8^**^	0.53 ± 0.33	181.2 ± 34.0	248.5 ± 20.9	4.70 ± 0.54
	108 mg/kg	56.7 ± 4.7^**^	169.5 ± 26.9^**^	0.48 ± 0.29	208.3 ± 32.9	268.0 ± 22.1	5.17 ± 0.59^*^
7-day	36 mg/kg	39.5 ± 7.2	131.3 ± 22.6	0.57 ± 0.34	145.7 ± 37.7^*^	236.4 ± 16.3^*^	4.79 ± 0.46
	72 mg/kg	42.8 ± 6.2	152.5 ± 47.2	0.63 ± 0.32	177.8 ± 45.6	264.3 ± 41.0	4.63 ± 0.44
	108 mg/kg	48.3 ± 4.4^**^	143.5 ± 13.7^**^	1.43 ± 0.43^**^	185.0 ± 39.6	278.0 ± 25.8	4.35 ± 0.38
14-day	36 mg/kg	49.0 ± 8.8^*^	182.3 ± 78.1	1.30 ± 0.60^**^	163.3 ± 68.4	270.7 ± 20.0	4.26 ± 0.66
	72 mg/kg	56.8 ± 4.7^**^	174.2 ± 51.6^*^	1.37 ± 0.30^**^	280.3 ± 82.8^*^	277.2 ± 38.3	4.88 ± 1.08
	108 mg/kg	52.8 ± 6.5^**^	172.2 ± 55.1^*^	1.43 ± 0.24^**^	148.8 ± 25.4^*^	265.1 ± 26.4	4.75 ± 0.75
21-day	36 mg/kg	54.8 ± 10.5^**^	169.7 ± 64.6	2.25 ± 0.79^**^	126.5 ± 46.0^*^	283.2 ± 35.4	4.18 ± 0.67
	72 mg/kg	46.2 ± 4.5^*^	128.5 ± 38.3	3.03 ± 0.82^**^	122.3 ± 13.4^**^	275.0 ± 31.0	4.59 ± 0.87
	108 mg/kg	58.3 ± 7.8^**^	176.0 ± 60.8^*^	1.95 ± 0.51^**^	144.2 ± 35.5^*^	302.8 ± 29.8	4.42 ± 0.30

Data was presented as mean ± SD, unless otherwise specified. *p* values were calculated by hypothesis testing. Data distribution was assessed by the Shapiro-Wilk test. Bilateral student’s t-test was used for normally distributed data. While the Mann-Whitney *U* test was used for nonparametric data. *, *p* < 0.05; **, *p* < 0.01.

### Overview of the data quality achieved from targeted metabolomics analysis

A targeted metabolomics method was used for the metabolites quantification in serums samples harvested from the animal experiment. A total of 289 metabolites with important biological significance were detected in a single-injection using both positive and negative modes with rapid polarity switching. To improve the sensitivity of metabolite detection, scheduled multiple reaction monitoring (s-MRM) mode was employed for metabolites quantification. The limits of quantification (LOQs) of metabolites ranged from 0.02 to 100 ng/ml, with over 90% of them lower than or equal to 10 ng/ml. The good sensitivity achieved in this targeted metabolomics method guaranteed the successful detection of endogenous metabolites with low abundance.

**FIGURE 2:**
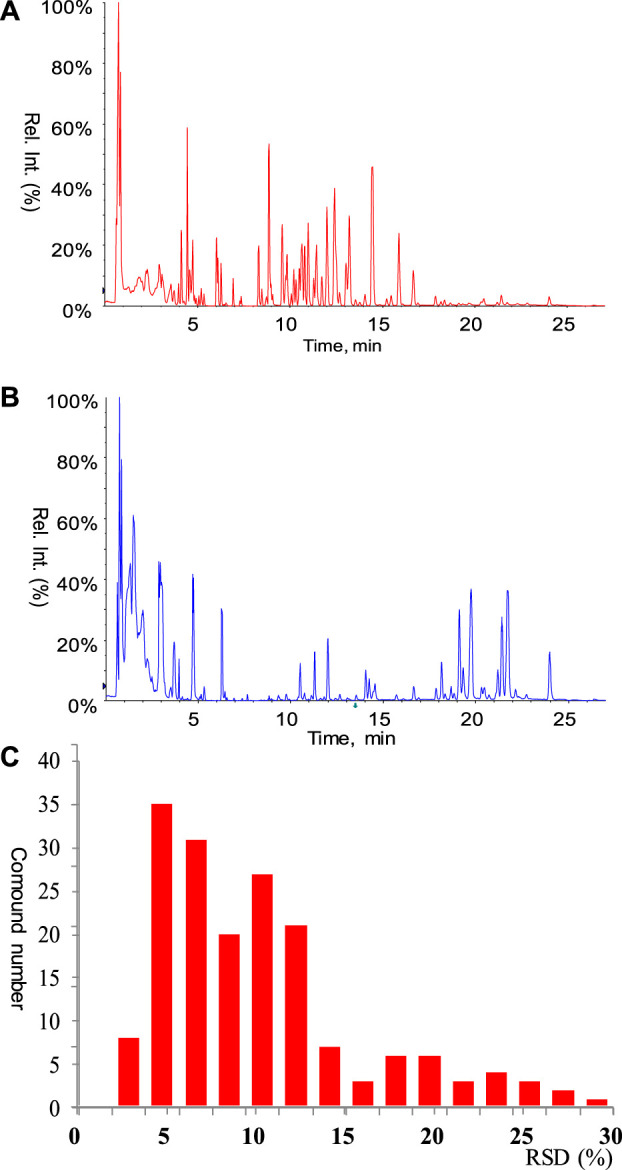
**(A)** Chromatogram of mixed standard. All the standards involved in the calibration curves were listed in Supplementary Table S1. **(B)** Chromatogram of rat serum. **(C)** Performance of pooled serum QC samples.

Finally, a total of 205 metabolites were quantified in rat serum samples. Chromatograms of the standards and rat serum were shown in Figures 2A,B, respectively. Pooled serum samples were used as quality controls (QCs) and evenly interspersed throughout the running batch, with one QC sample inserted after every nine tested samples. The coefficient of variation (CV) of all the QC samples were calculated to investigate the deviation introduced from sample pretreatment and instrumental analysis, with result shown in Figure 2C. CV values of all the metabolites in QC samples were less than 30%, with 89% of them below 20%. The good performance of QC samples suggested that the targeted metabolomics data obtained in this analytical batch was reliable and can be further submitted to statistical analysis.

### Multivariate analysis of the targeted metabolomics data

**FIGURE 3:**
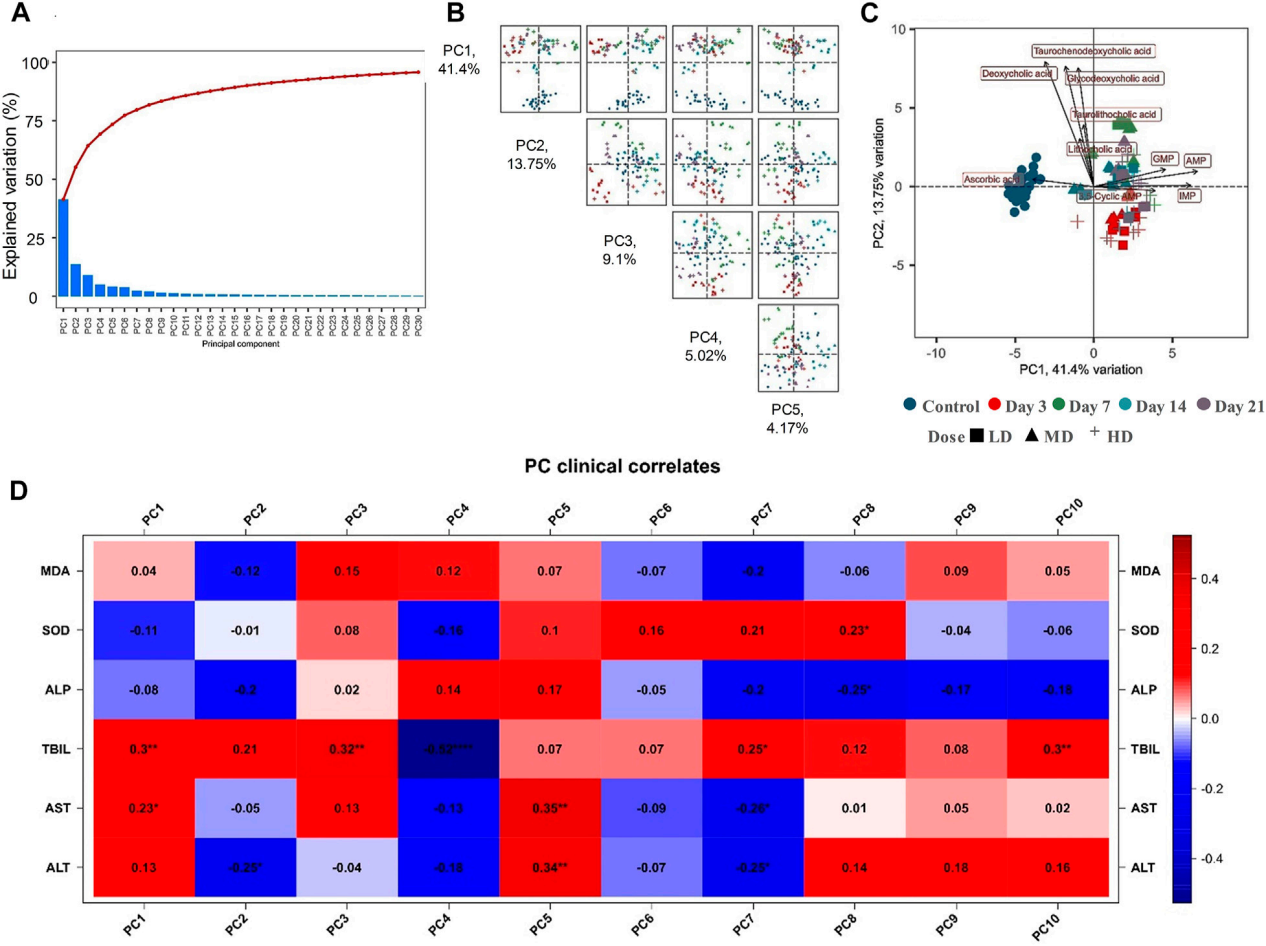
**(A)** The top 30 PCs generated from the PCA analysis based on the targeted metabolomics analysis, together with their explained variation values. **(B)** Pairs plot of the PCA score scatter plots using the top 5 PCs. **(C)** PCA score scatter plot generated from the top two PC2. **(D)** Correlations between the serum biochemical indicators and the PCs.

Multivariate analysis was conducted to investigate the differences of metabolomics phenotypes among different moxifloxacin administration groups. Moxifloxacin administration groups with different dosages and different administration times were all applied for PCA analysis based on the metabolites concentrations. Results was shown in Figure 3. The top 30 principal components (PCs) were listed in Figure 3A, together with their corresponding variance explanation rates. Figure 3B showed a pairs plot of the PCA score scatter plots using the top 5 PCs. The variance explanation rates of these 5 PCs were 41.4%, 13.75%, 9.1%, 5.02% and 4.17%, respectively. The cumulative variance explanation rate of the top 5 PCs was 73.44%, with the top 2 PC explained over 55% of the variation. A PCA score scatter plot generated by using PC1 and PC2 was shown in Figure 3C. As shown in the figure, the control group was clearly separated from the moxifloxacin administration groups. For the dosing groups, the samples were segregated into tight clusters according to the duration of moxifloxacin administration. Moxifloxacin administration time has a more significant effect on serum metabolome than administration dosage. Among the 3, 7, 14 and 21-day continuous dosing groups, the 7-day administration group showed the greatest metabolic difference from the control group.

The correlations between each PCs and the serum biochemical indicators were also explored based on Spearman’s rank correlation analysis, with result shown in Figure 3D. The liver function related biochemical indicators, including TBIL, AST and ALT, possessed the most obvious correlation with the PCs. TBIL showed a significant positive correlation (*p* < 0.05) with PC1, PC3, PC7, and PC10. While significant negative correlation was observed between control TBIL and PC4. AST was positively correlated with PC1 and PC5, and negatively correlated with PC7. ALT was positively correlated with PC5, and negatively correlated with PC2 and PC7.

### Dose-dependent manners of moxifloxacin related hepatotoxicity

Through comprehensive analysis of the pathological section analysis and the multivariate analysis of the targeted metabolomics data, it was demonstrated that the most serious liver injury related to moxifloxacin occurred in the 7-day administration group. Three different dosages (LD, MD and HD) of moxifloxacin were set up in the 7-day administration group. Dose-dependent manners of moxifloxacin related hepatotoxicity were investigated by using the targeted metabolomics data of the 7-day administration group with different dosages.

**FIGURE 4:**
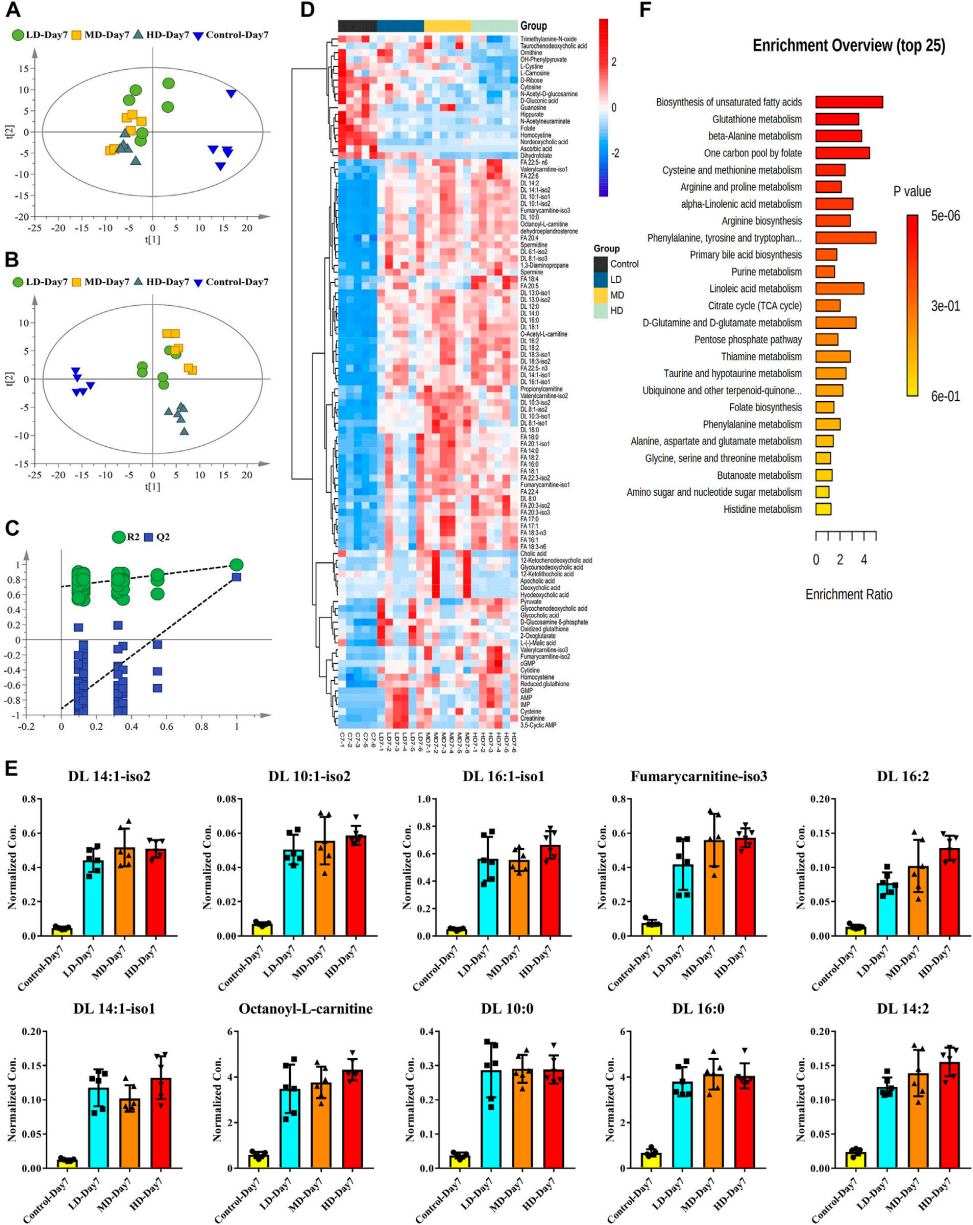
Dose-dependent manners of moxifloxacin related hepatotoxicity investigated by using the targeted metabolomics data of the 7-day administration groups. **(A)** PCA score scatter plot. **(B)** PLS-DA score scatter plot. **(C)** Random permutation test of the PLS-DA model. **(D)** Heatmap of the significantly changed metabolites between control group and 7-day HD group. **(E)** Dose-dependent manners of the top 10 fatty acyl carnitines with the most significant changes between the control group and the HD group. **(F)** Metabolic pathways associated with moxifloxacin related hepatotoxicity.

Both PCA and PLS-DA were applied to integrate all the metabolites detected in rat serum to investigate the dose-dependent manners of moxifloxacin. The score scatter plots of PCA and PLS-DA were shown in Figures 4A,B, respectively. No overfitting of the PLS-DA model was observed through a random permutation test with 100 iterations (Figure 4C). The moxifloxacin administration groups were clearly separated from the control group in the unsupervised PCA analysis (Figure 4A). While the distinction among LD, MD and HD groups was less pronounced in the multivariate statistical analysis. The HD group exhibited the most obvious difference from the control group (Figure 4B). Bilateral T-test and fold change (FC) analysis were further carried out to sieve the most obviously changing metabolites between the control group and 7-day HD group. A total of 101 metabolites were found to be significantly changed between control group and 7-day HD group, with adjust *p* value less than 0.05 and FCs larger than 2 (Supplementary Table S2).

To visualize the level of each metabolite in every sample, concentrations of the significantly changed metabolites were presented as heat maps (Figure 4D). Almost all the fatty acyl carnitine and free fatty acids were significantly up-regulated in the 7-day moxifloxacin administration group. Dose-dependent manners of the top 10 fatty acyl carnitines with the most significant changes between the control group and the HD group were shown in the histograms of Figure 4E. Significant higher levels of fatty acyl carnitines were observed in the 7-day moxifloxacin administration groups compared to the control group. Among the LD group, MD group, and HD group, most of the fatty acyl carnitines were up-regulated in a dose-dependent manner. However, different bile acids showed different trends in the administration group (Figure 4D). Besides nordeoxycholic acid and taurochenodeoxycholic acid, which were significantly decreased, most of the bile acids were significantly up-regulated in the moxifloxacin administration group compared to the control group. Dehydroepiandrosterone is a steroid hormone that has been reported to be closely related to lipid metabolism (Qin et al., 2020; Teixeira et al., 2020; Yokokawa et al., 2020). It was also up-regulated in a dose-dependent manner in the 7-day moxifloxacin administration groups (Figure 4D and Supplementary Figure S1A).

All the significantly changed metabolites were subjected to MetaboAnalyst 5.0 for pathway enrichment analysis, with result shown in Figure 4F. The top 5 metabolic pathway related to moxifloxacin induced hepatotoxicity were biosynthesis of unsaturated fatty acids, glutathione metabolism, beta-alanine metabolism, one carbon pool by folate, and cysteine and methionine metabolism.

### Time-dependent manners of moxifloxacin related hepatotoxicity

Time-dependent manners of moxifloxacin related hepatotoxicity were investigated by using the targeted metabolomics data of the HD groups with different moxifloxacin administration time. The serum metabolomics differences between the control group and the HD administration group at different dosing time points were first investigated.

**FIGURE 5:**
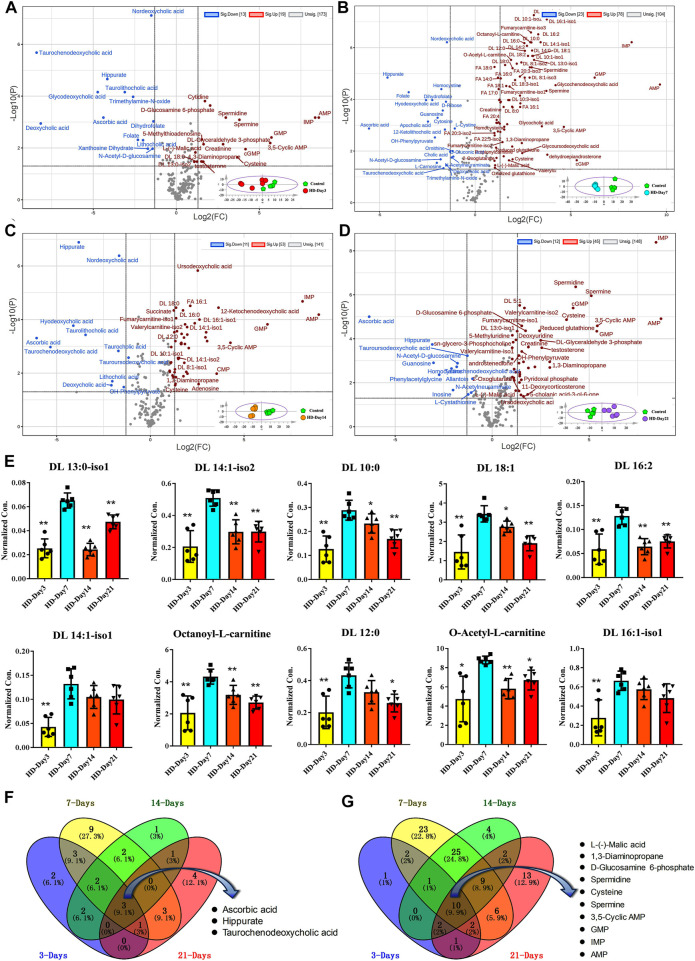
**(A–D)** Volcano plots based on the targeted metabolomics data of control group and HD group in the 3-day, 7-day, 14-day and 21-day dosing durations, respectively. **(E)** Time-dependent manners of the top 10 fatty acyl carnitines with the most significant changes among different moxifloxacin dosing durations. Statistical differences between the other HD groups ad the 7-day HD group were examined using t-tests. *, *p* < 0.05; **, *p* < 0.01. **(F,G)** Venn plots showing the overlap of the significantly decreased and increased metabolites in HD group of different dosing durations, respectively.

Volcano plots based on the targeted metabolomics data of control group and HD group in the 3-day, 7-day, 14-day and 21-day administration time points were shown in Figures 5A–D, respectively. The corresponding PCA plots were shown in the lower right corner of the volcano plots. Metabolites satisfying both adjust *p* value less than 0.05 and FCs larger than 2 were considered as compounds that varied significantly between the control group and HD group. According to this criterion, there were 32, 101, 64 and 57 metabolites were significantly changed between the HD group and control group in the 3-day, 7-day, 14-day, and 21-day dosing durations, respectively. By comparing the volcano plots for different moxifloxacin dosing durations, serum metabolomics perturbations were most pronounced in the 7-day dosing groups, which was consistent with the result of pathological analysis. Significant increases in fatty acyl carnitines and fatty acids were occurred in the 7-day HD group compared to the control group. However, the differences in fatty acyl carnitine and fatty acid levels between HD group and control group gradually became insignificant at 14-day and 21-day of administration. A heatmap of the significantly changed metabolites among the HD groups with different dosing durations were shown in Supplementary Figure S2. Time-dependent manners of the top 10 fatty acyl carnitines with the most significant changes among different moxifloxacin dosing durations were shown in the histograms of Figure 5E. Among HD groups with different dosing durations, the 7-day HD group showed the highest levels of fatty acyl carnitines. Besides, dehydroepiandrosterone was also found to be significantly changed among the HD groups with different dosing durations, with the highest concentrations in the 7-day HD group (Supplementary Figure S1B). According to the result of pathological analysis, the most serious liver injury related to moxifloxacin also occurred in the 7-day HD group. Changes in fatty acyl carnitines and dehydroepiandrosterone at different dosing durations were believed to be closely related to the hepatotoxicity of moxifloxacin.

Venn plots showing the overlap of the significantly decreased and increased metabolites in HD group of different dosing duration were shown in Figures 5F,G, respectively. Three metabolites were significant down-regulated in the HD group at four different dosing durations, including hippuric acid, taurochenodeoxycholic acid, and ascorbic acid (Figure 5F). While ten metabolites were significantly up-regulated in the HD group at different dosing durations, including AMP, IMP, GMP, 3′, 5′-cyclic AMP, cysteine, spermine, spermidine, D-glucosamine 6-phosphate, 1,3-diaminopropane, and L-malic acid (Figure 5G). These compounds that changed significantly over different dosing durations should be metabolic changes associated with the efficacy of moxifloxacin.

### Correlation analysis of the metabolites and biochemical indicators

**FIGURE 6:**
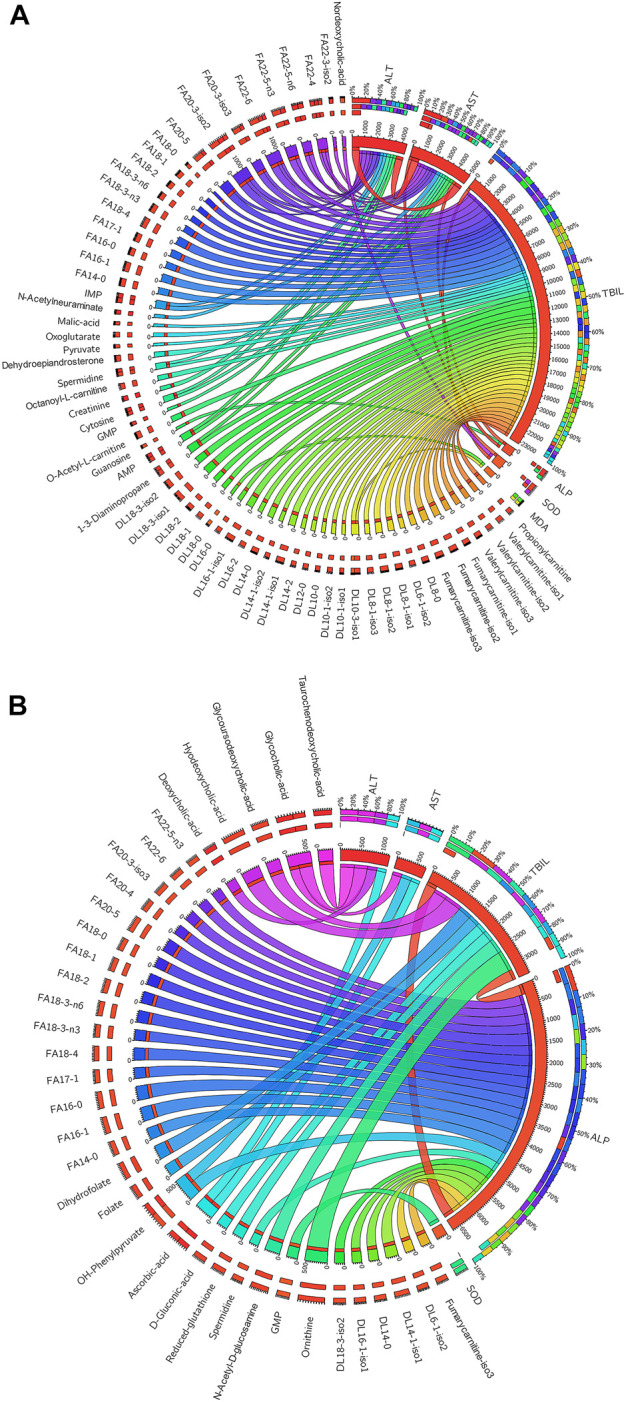
**(A)** Significant positive (*p* < 0.05) correlations existed between the metabolites and biochemical indicators. **(B)** Significant negative correlations existed between the metabolites and biochemical indicators (*p* < 0.05). The thicker the line, the stronger the correlation.

Since the organism presents a complex network, a correlation analysis can well reflect the internal relationship between the metabolites and the liver function related biochemical indicators. The metabolites used for the correlation analysis were the 101 compounds that changed signficntly between the control group and the HD group at the 7-day dosing time point. The biochemical indicatiors were ALT, AST, TBIL, ALP, SOD and MDA (Table 1). Spearman’s rank correlation analysis was performed between metabolites and biochemical indicators. Only significant correlations with *p* value less than 0.05 were displayed in circos plots (Figure 6). The TBIL presented positive correlations with the vast majority of fatty acyl carnitines and fatty acids (Figure 6A). ALT and AST also presented significant postive correlations with the long chain fatty acids (carbon number greater than 18). As shown in Figure 6B, ALP presented significant negative correlations with the fatty acyl carnitines and fatty acids. While, ALT, AST and TBIL were negatively correlated with the bile acids.

## Discussion

Moxifloxacin is an antibiotic drug that widely used in clinic with good curative effect (Balfour and Wiseman, 1999). However, the rare and severe liver injury induced by moxifloxacin was a big concern in clinical practice (Soto et al., 2002). Statistic showed that the liver toxicity caused by moxifloxacin was more than twice as high as amoxicillin-clavulanic acid (Adminstration, 2006). Moxifloxacin is one of the most common drugs that causes DILI in clinic (Paterson et al., 2012). Early warning and detection of liver toxicity caused by moxifloxacin are critical for reducing clinical adverse effect.

Pathological analysis of liver tissue demonstrated that moxifloxacin administration could cause hepatotoxicity accompanied by hepatocellular apoptosis and necrosis in a dose-dependent manner (Figure 1). The liver injury caused by moxifloxacin can be occurred in short time. A continuous administration of moxifloxacin for 3- to 7-day can cause transient liver damage, with the most significant liver injury occurred in the 7-day HD group (Figure 1). However, the liver injury caused by moxifloxacin did not worsen with the prolongation of administration time. Only mild hepatotoxicity was observed in the 14-day moxifloxacin administration group. The live injury was almost automatic recovery in the 21-day administration group. According to reports in the literature, the main characteristics of moxifloxacin induced liver injury were the short latency and abrupt onset of injury (Orman et al., 2011; Paterson et al., 2012). The clinical presentation and pattern of DILI are similar for the various fluoroquinolones, consistent with a “class effect” (Fisher et al., 2015). DILI related to fluoroquinolones is characterized by a short latency period of 2–9 days in clinical practice (Fisher et al., 2015). The median time from the fluoroquinolone use to the earliest onset of DILI was 4 days, and the median time to abnormal liver function was 8.5 days (Orman et al., 2011). The hepatotoxicity of fluoroquinolones appears to be an immune-mediated DILI as evidence by the short latency period (Kaplowitz, 2005; Schmid et al., 2006; Abboud and Kaplowitz, 2007; Orman et al., 2011; Katarey and Verma, 2016). Most of the fluoroquinolone-induced hepatotoxicity could recover after drug withdrawal (Fisher et al., 2015). The results of this study demonstrated that moxifloxacin can cause transient liver injury, which was most severe on the seventh day of continuous administration. The liver injury related to moxifloxacin can automatically restored over time without drug withdrawal in the current study, which was a very interesting phenomenon and deserved further study.

DILI can be classified as hepatocellular, cholestatic, or mixed on the basis of the biochemical indicators based on the ratio of ALT to ALP (R value) in serum (Fisher et al., 2015). DILI may present as cholestatic pattern (Giannini et al., 2005). The cholestatic damage pattern is characterized by ALP greater than 3 times the upper limit of normal (ULN) and/or R < 2. As shown in Table 1, the increase of serum ALT, AST and ALP of the dosing groups were all less than 2 times of the values in control group, and they did not change in a time- or dose-dependent manner.

Bilirubin is a yellowish substance that releases from the destroyed red blood cells and passes on to the liver (Woreta and Alqahtani, 2014). The bilirubin will not be properly released when the liver is not functioning correctly (Woreta and Alqahtani, 2014; Weaver et al., 2018). Total bilirubin is the sum of direct and indirect bilirubin. If the TBIL level is higher than expected, the liver may have become dysfunctional (Woreta and Alqahtani, 2014). As shown in Table 1, the serum levels of TBIL increased significantly with the extension of moxifloxacin administration time. Hyperbilirubinemia is a characteristic of the cholestatic liver disease. According to the previous research, increased bilirubin concentration was a relatively late event in chronic liver diseases (Fevery, 2008). In this study, the changes of TBIL also lagged behind AST and ALT. From the perspective of serum biochemical indices related to liver function, DILI caused by moxifloxacin is most likely to be cholestatic pattern. Unfortunately, the conventional liver function-related biochemical indicators have limited sensitivity in predicting liver injury induced by moxifloxacin.

Targeted metabolomics is defined as the full characterization of specific small molecule metabolites, which has been emerged as a useful tool for the exploration of pathological mechanisms and diagnostic markers of diseases (Johnson et al., 2016). However, the metabolomic disturbance related to moxifloxacin induced liver injury was rarely investigated. Here, targeted metabolomics was conducted to illustrate the dose-and time-dependent manners of moxifloxacin related hepatotoxicity. Fatty acyl carnitines and fatty acids were found to change dynamically with the severity of liver injury induced by moxifloxacin (Figures 4, 5). They were significantly elevated when liver injury was severe, and their concentrations decreased with recovery from liver injury. They were supposed to play important roles in the pathological process of liver injury. The serum concentrations of fatty acyl carnitines and fatty acids have good prospects in predicting moxifloxacin induced liver injury.

The liver plays central role in lipid metabolism by absorbing serum free fatty acids, and manufacturing, storing, and transporting lipid metabolites (Musso et al., 2009). Elevated fatty acid levels in the liver can cause hepatocyte injury via the formation of various toxic metabolites, such as eicosanoids, ceramide, diacylglycerols, and lysophosphatidylcholine (Bessone et al., 2019; Wang et al., 2021). Some resent studiers have found that fatty acid accumulation in the liver tissue may be an important cause of hepatotoxicity for patients with metabolic syndrome (McClain et al., 2007; Yamaguchi et al., 2007). Hepatocytes, exposure to high levels of serum free fatty acids, and stored FFAs in the form of triglycerides may ease FFA-induced oxidative stress and chronic inflammation, thereby preventing further hepatocellular damage (Liu et al., 2016). Some *in vivo* and *in vitro* studies suggested that hepatic triglycerides deposition may not be harmful; rather, it may be a self-protection measure of the organism against fatty acid induced lipotoxic liver injury by storing fatty acids in the form of triglycerides (Listenberger et al., 2003; McClain et al., 2007; Yamaguchi et al., 2007; Liu et al., 2016).

Carnitine, primarily of dietary origin, participates in the transport of long-chain fatty acids to mitochondria for subsequent β-oxidation. Acyl carnitine is acyl esters of the carnitine. It is essential for the catabolism of fatty acids and maintenance of energy homeostasis in the human body. Acyl carnitine transport acyl groups from fatty acids into the mitochondria to generate energy. These molecules are capable of moving through the mitochondrial and cell membranes via a carnitine shuttle. Therefore, fatty acyl carnitines are direct precursors for the oxidation of long-chain fatty acids. In this study, the concentrations of serum fatty acyl carnitines were significantly increased with the severity of hepatotoxicity induced by moxifloxacin, together with the levels of fatty acids. These results indicated that both the serum concentrations of fatty acids and the oxidative efficiency of fatty acids were significantly increased under the pathological condition of liver injury induced by moxifloxacin. The oxidation of fatty acids in mitochondria of the liver cells is one of the main sources of ROS (Peralta et al., 2013). The elevated fatty acid and fatty acyl carnitines in the moxifloxacin dosing groups could resulted in the overproduction of ROS, such as hydroxyl radical, hydrogen peroxide, and superoxide anion. ROS can inhibit or deplete endogenous enzymatic and nonenzymatic antioxidants, cause oxidative stress, and lead to liver cell apoptosis and liver lipid peroxidation (Xu et al., 2018).

Besides the fatty acyl carnitines and fatty acids, dehydroepiandrosterone was also proved to change dynamically with the severity of moxifloxacin induced hepatotoxicity. Pearson’s correlation analysis indicated that dehydroepiandrosterone was significantly correlated with fatty acyl carnitine and fatty acids (Supplementay Table S3). Dehydroepiandrosterone was a natural steroid hormone produced from cholesterol, which was believed to play important roles in the metabolism of lipid compounds (Cheng et al., 2011; Espinosa De Ycaza et al., 2016; Teixeira et al., 2020). Hepatocytes treated by dehydroepiandrosterone exhibited a decline in acyl carnitines, which declined the catabolism of fatty acid and suppressed the generation of reactive oxygen species (ROS) (Tang et al., 2007; Abd-Ella et al., 2020). Dehydroepiandrosterone was proved to protect against acetaminophen induced liver damage in rats (Abd-Ella et al., 2020). It is clear that dehydroepiandrosterone plays a protective role against liver injury (Kuebler et al., 2001; Aoki et al., 2003; Abd-Ella et al., 2020). In the current study, the serum levels of dehydroepiandrosterone were significantly up-regulated with the severity of hepatotoxicity induced by moxifloxacin. The researchers speculate that this is a feedback regulation mechanism. With the increase of fatty acyl carnitines and fatty acids in serum after liver injury, the body reduces the lipotoxicity by increasing dehydroepiandrosterone levels, and thus plays a role in liver protection.

The glutathione metabolism was found to be one of the most obviously disturbed metabolic pathways related to live injury induce by moxifloxacin. Oxidized glutathione, cysteine, spermidine and spermine were all significantly up-regulated in the moxifloxacin adminstration group (Figure 4D). As the core metabolite of glutathione metabolism pathway, glutathione is a tripeptide containing γ-amide bonds and sulfhydryl groups. It is compounded of glutamate, cysteine, and glycine (Gerard-Monnier and Chaudiere, 1996; Dominko and Dikic, 2018). Glutathione metabolic pathway is the most important metabolic pathway related to detoxification in the organism. Glutathione is the most abundant cellular thiol antioxidant and it exhibits numerous and versatile function (Gerard-Monnier and Chaudiere, 1996; Arthur, 2000). It can directly scavenge the ROS, and serve as a cofactor the enzyme glutathione peroxidase in metabolizing hydrogen peroxide and lipid peroxides (Arthur, 2000; Chen et al., 2013). In this study, the up-regulation of glutathione and related metabolites in the dosing group can be a self-protective measure against the hepatotoxicity of the moxifloxacin.

## Conclusion

**FIGURE 7:**
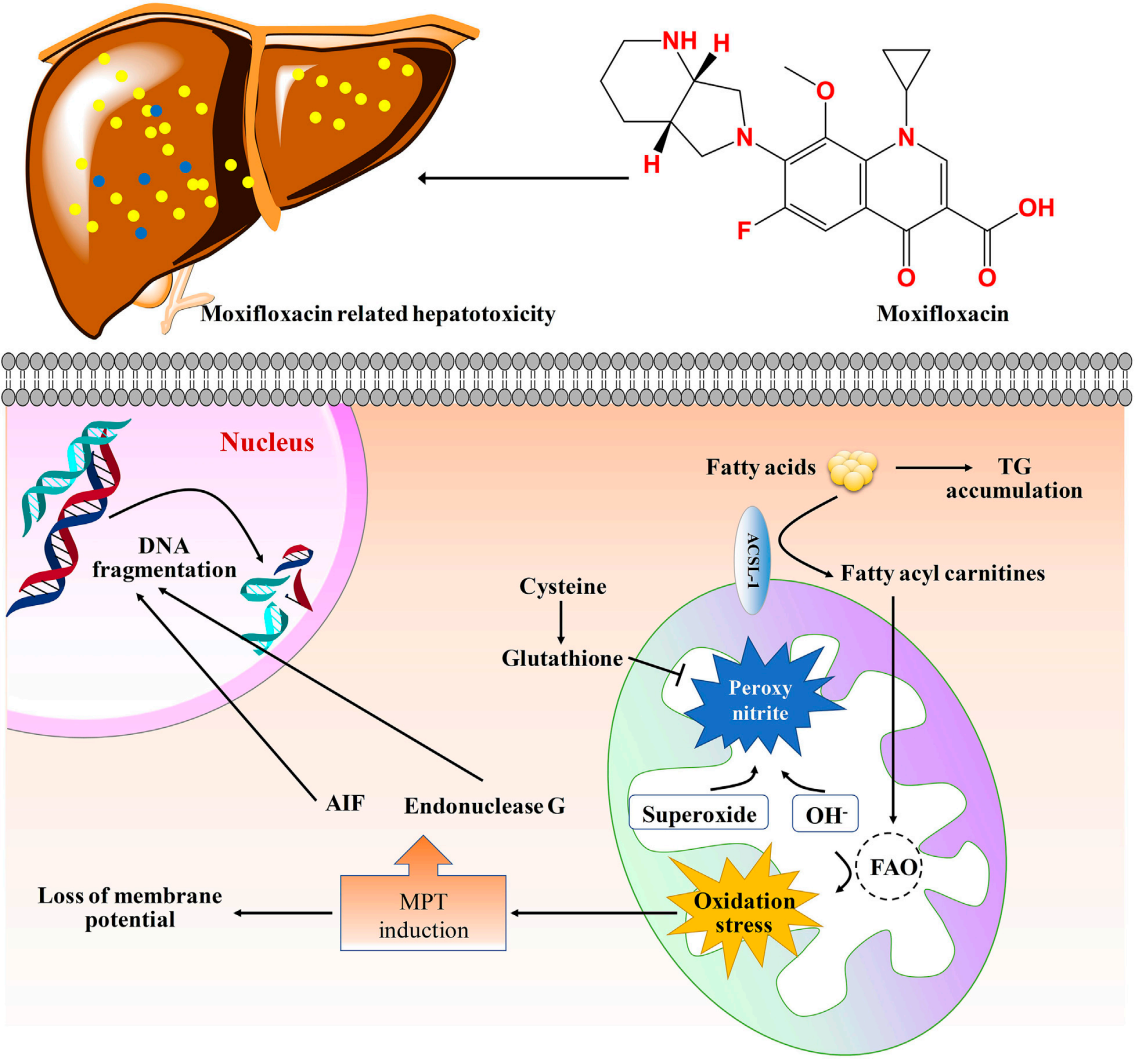
Molecular network diagram of the toxic and side effects of moxifloxacin on the liver tissue. Moxifloxacin administration could up-regulated the levels of fatty acyl carnitines, thus increased the generation of ROS. The generation of ROS will cause damage to biological macromolecules. The MPT (mitochondrial permeability transition) triggers matrix swelling with rupture of the outer member and release of intermembrane proteins including apoptosis-inducing factor (AIF) and endonuclease G, which translocate to the nucleus and cause nuclear DNA fragmentation (Woolbright and Jaeschke, 2017). GSH can scavenge ROS and peroxynitrite in mitochondria. TG, triglycerides; FAO, fatty acid oxidation.

In conclusion, this study disclosed the dose- and time-dependent manners of moxifloxacin induced liver injury. Pathological analysis showed that moxifloxacin can cause obvious transient liver damage in a short period of time, and the liver injury was most serious when the drug was used continuously for about 7 days. After that, the transient liver injury can be gradually heal on its own without any treatment. The hepatotoxicity related to moxifloxacin is most likely to be cholestatic pattern based on the changes of serum biochemical indicators. However, the conventional liver function related biochemical indicators can hardly be used for the early warning of liver injury caused by moxifloxacin due to their limited sensitivity and significant hysteresis. Fatty acyl carnitines, fatty acids and dehydroepiandrosterone were found to change dynamically with the severity of moxifloxacin related hepatotoxicity by targeted metabolomics analysis. The results achieved in this study demonstrated the broad prospects of elevated serum fatty acyl carnitines, fatty acids and dehydroepiandrosterone in predicting moxifloxacin induced liver injury. A molecular network diagram of the toxic and side effects of moxifloxacin on the liver based on your own results and literature was shown in Figure 7.

## Data Availability

The raw data supporting the conclusion of this article will be made available by the authors, without undue reservation.
