# Supplementary material for: Dose- and time-dependent manners of moxifloxacin induced liver injury by targeted metabolomics study
Source: Front Pharmacol. 2022 Sep 16;13:994821. doi: 10.3389/fphar.2022.994821 (PMC9525095; doi:10.3389/fphar.2022.994821)
Supplement: Supplementary file 1 [file DataSheet1.zip › supplementary materials/Figure S1.docx]

**Figure S1.** (A) Dose-dependent manner of dehydroepiandrosterone. (B) Time-dependent manner of dehydroepiandrosterone. Statistical differences between the other HD groups and the 7-day HD group were examined using t-tests. *, P<0.05; **, P<0.01.
