# Supplementary material for: Dose- and time-dependent manners of moxifloxacin induced liver injury by targeted metabolomics study
Source: Front Pharmacol. 2022 Sep 16;13:994821. doi: 10.3389/fphar.2022.994821 (PMC9525095; doi:10.3389/fphar.2022.994821)
Supplement: Supplementary file 1 [file DataSheet1.zip › supplementary materials/Figure S2.docx]

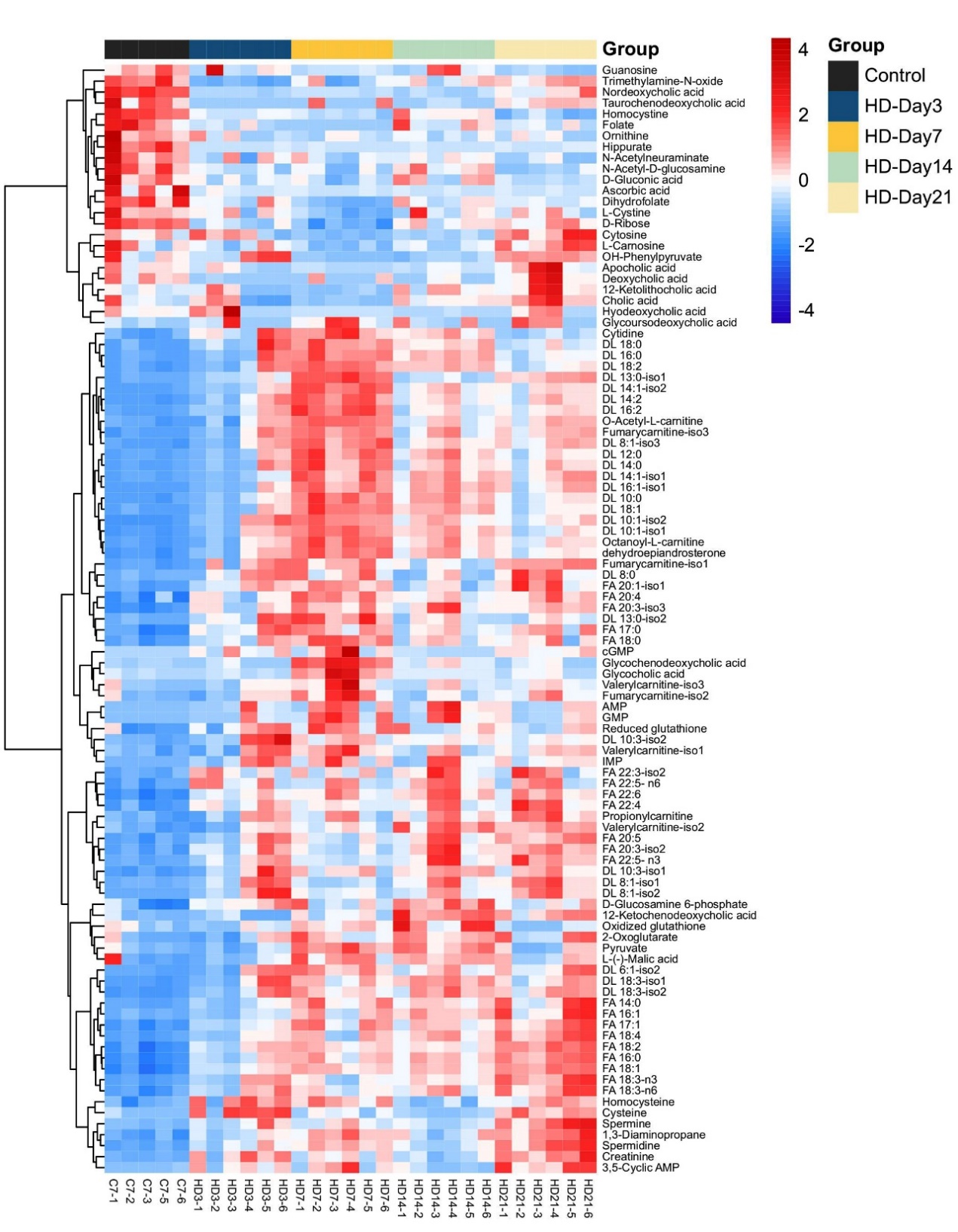


**Figure S2.** Heatmap of the significantly changed metabolites among the HD groups with different moxifloxacin dosing durations.
