# Supplementary material for: Dose- and time-dependent manners of moxifloxacin induced liver injury by targeted metabolomics study
Source: Front Pharmacol. 2022 Sep 16;13:994821. doi: 10.3389/fphar.2022.994821 (PMC9525095; doi:10.3389/fphar.2022.994821)
Supplement: Supplementary file 1 [file DataSheet1.zip › supplementary materials/Table S1.docx]

**Table S1.** Metabolites information and MRM parameters.

| **Metabolites** | **CAS NO.** | **Formula** | **MS polarity** | **Q1** | **Q3** | **DP** | **CE** |
| --- | --- | --- | --- | --- | --- | --- | --- |
| Choline | 62-49-7 | C5H14NO^+^ | Positive | 104.1 | 60 | 90 | 40 |
| Niacinamide | 98-92-0 | C6H6N2O | Positive | 123.1 | 80 | 70 | 30 |
| 1,3-Diaminopropane | 109-76-2 | C3H10N2 | Positive | 75 | 58 | 112 | 15 |
| Glycerol | 56-81-5 | C3H8O3 | Positive | 93 | 66 | 207 | 21 |
| L-Glutamine | 56-85-9 | C5H10N2O3 | Positive | 147 | 84 | 140 | 35 |
| L-Histidine | 71-00-1 | C6H9N3O2 | Positive | 156.1 | 110 | 70 | 20 |
| L-Proline | 147-85-3 | C5H9NO2 | Positive | 116 | 70 | 100 | 40 |
| L-Phenylalanine | 63-91-2 | C9H11NO2 | Positive | 166 | 120 | 90 | 18 |
| L-Glutamic acid monohydrate | 56-86-0 | C5H9NO4 | Positive | 148.1 | 84 | 60 | 38 |
| L-isoleucine | 73-32-5 | C6H13NO2 | Positive | 132 | 86 | 50 | 14 |
| L-Tyrosine | 60-18-4 | C9H11NO3 | Positive | 182 | 136 | 120 | 19 |
| L-Cystine | 56-89-3 | C6H12N2O4S2 | Positive | 241 | 152 | 80 | 19 |
| L-Alanine | 56-41-7 | C3H7NO2 | Positive | 90 | 44 | 104 | 20 |
| HEPES | 7365-45-9 | C8H18N2O4S | Positive | 239.2 | 131 | 200 | 30 |
| Thiamine pyrophosphate | 154-87-0 | C12H19ClN4O7P2S | Positive | 425.2 | 122 | 120 | 40 |
| 5'-Methylthioadenosine | 2457-80-9 | C11H15N5O3S | Positive | 298.1 | 136 | 90 | 27 |
| S-Adenosyl-L-methionine | 86867-01-8 | C15H25Cl3N6O5S | Positive | 399.4 | 298 | 100 | 12 |
| AMP | 61-19-8 | C10H14N5O7P | Positive | 348.1 | 136 | 80 | 70 |
| GMP | 7665-99-8 | C10H12N5O7P | Positive | 346.1 | 152 | 90 | 30 |
| dAMP | 653-63-4 | C10H14N5O6P | Positive | 332.1 | 136 | 60 | 22 |
| L-Carnosine | 305-84-0 | C9H14N4O3 | Positive | 227.3 | 122 | 100 | 19 |
| Homocysteine | 454-29-5 | C4H9NO2S | Positive | 136.1 | 90 | 60 | 15 |
| Guanosine | 118-00-3 | C10H13N5O5 | Positive | 284.2 | 152 | 60 | 20 |
| CMP | 63-37-6 | C9H14N3O8P | Positive | 324.1 | 112 | 60 | 25 |
| L-Aspartate | 56-84-8 | C4H7NO4 | Positive | 134 | 74 | 60 | 20 |
| Acetylcarnitine | 3040-38-8 | C9H17NO4 | Positive | 204.2 | 85 | 100 | 69 |
| L-Asparagine | 70-47-3 | C4H8N2O3 | Positive | 133.1 | 87 | 50 | 12 |
| L-Valine | 72-18-4 | C5H11NO2 | Positive | 118 | 72 | 115 | 14 |
| Histamine | 51-45-6 | C5H9N3 | Positive | 112 | 95 | 60 | 10 |
| L-Lysine | 56-87-1 | C6H14N2O2 | Positive | 147.1 | 84 | 65 | 35 |
| Glycine | 56-40-6 | C2H5NO2 | Positive | 76 | 30 | 30 | 20 |
| L-Leucine | 61-90-5 | C6H13NO2 | Positive | 132.1 | 86 | 100 | 20 |
| trans-4-Hydroxy-L-proline | 51-35-4 | C5H9NO3 | Positive | 132.1 | 86 | 100 | 19 |
| Thymidine | 50-89-5 | C10H14N2O5 | Positive | 243.1 | 127 | 80 | 20 |
| L-Serine | 56-45-1 | C3H7NO3 | Positive | 106 | 60 | 55 | 15 |
| Nicotinate | 59-67-6 | C6H5NO2 | Positive | 124.1 | 80 | 120 | 30 |
| Ornithine | 3184-13-2 | C5H12N2O2 | Positive | 133.1 | 70 | 100 | 22 |
| L-Tryptophan | 73-22-3 | C11H12N2O2 | Positive | 205.2 | 188 | 100 | 14 |
| sn-Glycero-3-phosphocholine | 28319-77-9 | C8H20NO6P | Positive | 258.2 | 104 | 100 | 22 |
| GMP | 85-32-5 | C10H14N5O8P | Positive | 364.1 | 152.1 | 60 | 28 |
| Trimethylamine-N-oxide | 1184-78-7 | C3H9NO | Positive | 76.1 | 42 | 160 | 34 |
| NG,NG-Dimethylarginine | 30315-93-6 | C8H18N4O2 | Positive | 203.2 | 158 | 120 | 20 |
| L-Kynurenine | 2922-83-0 | C10H12N2O3 | Positive | 209.2 | 94 | 40 | 20 |
| N-Acetyl-L-Glutamine | 2490-97-3 | C7H12N2O4 | Positive | 189.2 | 130 | 35 | 23 |
| Adenine | 73-24-5 | C5H5N5 | Positive | 135.8 | 118.8 | 60 | 20 |
| Allantoin | 97-59-6 | C4H6N4O3 | Positive | 159.1 | 116 | 60 | 11 |
| Aminoisobutyrate | 62-57-7 | C4H9NO2 | Positive | 104.1 | 58 | 100 | 17 |
| Glycylglycine | 556-50-3 | C4H8N2O3 | Positive | 133.1 | 76 | 70 | 13 |
| γ-Aminobutanoate | 56-12-2 | C4H9NO2 | Positive | 104.1 | 58 | 100 | 17 |
| Betaine | 107-43-7 | C5H11NO2 | Positive | 118.1 | 43 | 100 | 55 |
| Hypoxanthine | 68-94-0 | C5H4N4O | Positive | 137.1 | 110 | 160 | 28 |
| Creatine | 57-00-1 | C4H9N3O2 | Positive | 132.1 | 90 | 135 | 27 |
| Cysteine | 52-90-4 | C3H7NO2S | Positive | 122.2 | 59 | 45 | 30 |
| DL-Pyroglutamic acid | 149-87-1 | C5H7NO3 | Positive | 130.1 | 84 | 70 | 18 |
| Hippurate | 495-69-2 | C9H9NO3 | Positive | 180.1 | 105 | 60 | 20 |
| Cystamine | 51-85-4 | C4H12N2S2 | Positive | 153.2 | 108 | 90 | 25 |
| DOPA | 59-92-7 | C9H11NO4 | Positive | 198.2 | 152 | 100 | 20 |
| Citrulline | 372-75-8 | C6H13N3O3 | Positive | 176.1 | 70 | 70 | 31 |
| cAMP | 60-92-4 | C10H12N5O6P | Positive | 330.1 | 136 | 110 | 33 |
| Inositol | 87-89-8 | C6H12O6 | Positive | 181.1 | 139 | 145 | 15 |
| Uracil | 66-22-8 | C4H4N2O2 | Positive | 113 | 70 | 140 | 20 |
| Pantothenate | 137-08-6 | C18H32CaN2O10 | Positive | 220.1 | 90 | 80 | 20 |
| 7-Methylxanthine | 552-62-5 | C6H6N4O2 | Positive | 167.1 | 124 | 100 | 28 |
| 2'-Deoxyadenosine | 16373-93-6 | C10H15N5O4 | Positive | 252.3 | 94 | 80 | 61 |
| Pyridoxal 5'-phosphate | 41468-25-1 | C8H12NO7P | Positive | 248.2 | 150 | 100 | 27 |
| 5-Methyluridine | 1463-10-1 | C10H14N2O6 | Positive | 259.1 | 127 | 60 | 16 |
| 2'-Deoxycytidine | 951-77-9 | C9H13N3O4 | Positive | 228.1 | 112 | 50 | 20 |
| Deoxyuridine | 951-78-0 | C9H12N2O5 | Positive | 229.2 | 113 | 60 | 20 |
| Inosine | 58-63-9 | C10H12N4O5 | Positive | 269.1 | 137 | 60 | 23 |
| Carnitine | 541-15-1 | C7H15NO3 | Positive | 162.1 | 103 | 60 | 35 |
| 4-Aminobenzoate | 150-13-0 | C7H7NO2 | Positive | 138.1 | 94 | 80 | 18 |
| Hydroxyproline | 51-35-4 | C5H9NO3 | Positive | 132.1 | 86 | 60 | 17 |
| Pyridoxine | 65-23-6 | C8H11NO3 | Positive | 170 | 134.2 | 60 | 20 |
| Cytosine | 71-30-7 | C4H5N3O | Positive | 112 | 95 | 60 | 20 |
| Dopamine | 51-61-6 | C8H11NO2 | Positive | 154.1 | 119 | 105 | 29 |
| Creatinine | 60-27-5 | C4H7N3O | Positive | 114.1 | 86 | 140 | 22 |
| Cytidine | 65-46-3 | C9H13N3O5 | Positive | 244.1 | 112 | 70 | 24 |
| N-Acetyl-D-glucosamine | 7512-17-6 | C8H15NO6 | Positive | 222.1 | 138 | 50 | 21 |
| 5-Methylcytidine | 2140-61-6 | C10H15N3O5 | Positive | 258.1 | 126 | 65 | 24 |
| N,N-Dimethylglycine | 1118-68-9 | C4H9NO2 | Positive | 104.1 | 58 | 50 | 21 |
| Cysteamine | 60-23-1 | C2H7NS | Positive | 78.1 | 61 | 88 | 17 |
| Melatonin | 73-31-4 | C13H16N2O2 | Positive | 233.3 | 174 | 130 | 21 |
| Uridine | 58-96-8 | C9H12N2O6 | Positive | 245.1 | 113 | 60 | 20 |
| 1,7-Dimethylxanthine | 611-59-6 | C7H8N4O2 | Positive | 181.1 | 124 | 90 | 27 |
| Homocystine | 626-72-2 | C8H16N2O4S2 | Positive | 269.3 | 136 | 90 | 14 |
| (±)-Epinephrine | 51-43-4 | C9H13NO3 | Positive | 184.2 | 107 | 100 | 30 |
| N-Acetylornithine | 6205-08-9 | C7H14N2O3 | Positive | 175 | 69.8 | 60 | 30 |
| Cobalamin | 68-19-9 | C63H88CoN14O14P | Positive | 678.8 | 147 | 230 | 65 |
| 3-Methylxanthine | 1076-22-8 | C6H6N4O2 | Positive | 167.1 | 124 | 100 | 24 |
| L-Arginine | 74-79-3 | C6H14N4O2 | Positive | 175 | 69.8 | 60 | 30 |
| L-Threonine | 72-19-5 | C4H9NO3 | Positive | 120.1 | 74 | 31 | 15 |
| Oxidized glutathione | 27025-41-8 | C20H32N6O12S2 | Positive | 613.1 | 230.8 | 180 | 48 |
| L-homoserine | 16504-56-6 | C4H9NO3 | Positive | 120 | 60 | 100 | 23 |
| DL 8:0 | 25243-95-2 | C15H29NO4 | Positive | 288.2 | 85 | 60 | 20 |
| L-Cystathionine | 56-88-2 | C7H14N2O4S | Positive | 223.2 | 134 | 100 | 21 |
| normetanephrine | 1011-74-1 | C9H14ClNO3 | Positive | 184.2 | 134 | 80 | 24 |
| Ala-Gly | 3695-73-6 | C5H10N2O3 | Positive | 147.1 | 90 | 100 | 19 |
| Adenosine | 131-99-7 | C10H13N5O4 | Positive | 268.1 | 136 | 70 | 30 |
| DCTP | 2056-98-6 | C9H16N3O13P3 | Positive | 468.1 | 395 | 60 | 15 |
| Putrescine | 110-60-1 | C4H12N2 | Positive | 89.1 | 72 | 100 | 23 |
| Spermidine | 124-20-9 | C7H19N3 | Positive | 146.2 | 112 | 100 | 13 |
| Spermine | 71-44-3 | C10H26N4 | Positive | 203.2 | 112.1 | 60 | 29 |
| Thiamine Hydrochloride (B1) | 70-16-6 | C12H17N4OS^+^ | Positive | 265.3 | 122 | 100 | 52 |
| Aldosterone | 52-39-1 | C21 H28 O5 | Positive | 361.2 | 343 | 170 | 26 |
| 11-Deoxycortisol | 152-58-9 | C21 H30 O4 | Positive | 347.2 | 97 | 170 | 33 |
| 11-Deoxycorticosterone | 64-85-7 | C21 H30 O3 | Positive | 331.2 | 97 | 170 | 27 |
| Corticosterone | 50-22-6 | C21 H30 O4 | Positive | 347.2 | 121.1 | 100 | 33 |
| Dehydroepiandrosterone (DHEA) | 53-43-0 | C19 H28 O2 | Positive | 289.2 | 85 | 100 | 27 |
| Cortisol | 50-23-7 | C21 H30 O5 | Positive | 363.2 | 121 | 180 | 32 |
| Dihydrotestosterone | 521-18-6 | C19H30O2 | Positive | 291.2 | 145 | 70 | 23 |
| Androstenedione | 63-05-8 | C19H26O2 | Positive | 287.2 | 97 | 170 | 30 |
| Testosterone | 58-22-0 | C19H28O2 | Positive | 289.2 | 109 | 100 | 32 |
| 4-Pyridoxic acid | 82-82-6 | C8H9NO4 | Positive | 184 | 148 | 60 | 40 |
| Reduced glutathione | 70-18-8 | C10H17N3O6S | Positive | 308 | 179 | 150 | 18 |
| Lactose | 63-42-3 | C12H22O11 | Negative | 341 | 89 | -120 | -25 |
| D-Gluconic acid | 133-42-6 | C6H12O7 | Negative | 195 | 129 | -80 | -27 |
| Taurine | 107-35-7 | C2H7NO3S | Negative | 124 | 80 | -100 | -32 |
| Fructose | 57-48-7 | C6H12O6 | Negative | 179.1 | 89 | -90 | -13 |
| Ascorbic acid | 50-81-7 | C6H8O6 | Negative | 174.9 | 86.8 | -60 | -20 |
| Oxoacetic Acid | 298-12-4 | C2H2O3 | Negative | 73 | 45 | -120 | -11 |
| Glucose | 50-99-7 | C6H12O6 | Negative | 179.1 | 89 | -90 | -13 |
| MOPS | 1132-61-2 | C7H15NO4S | Negative | 207.8 | 79.9 | -60 | -30 |
| Xylitol | 87-99-0 | C5H12O5 | Negative | 151 | 89 | -100 | -16 |
| Succinate | 150-90-3 | C4H4Na2O4 | Negative | 117 | 73 | -70 | -15 |
| DCMP | 13085-50-2 | C9H12N3Na2O7P | Negative | 306 | 79 | -120 | -50 |
| Pyridoxal phosphate | 853645-22-4 | C8H10NO6P | Negative | 246 | 79 | -120 | -60 |
| Thymidine 5′-monophosphate (DTMP) | 33430-62-5 | C10H13N2Na2O8P | Negative | 321.1 | 79 | -70 | -60 |
| Phosphoethanolamine | 1071-23-4 | C2H8NO4P | Negative | 140 | 79 | -80 | -30 |
| D-Glucosamine 6-phosphate | 3616-42-0 | C6H14NO8P | Negative | 258.1 | 79 | -120 | -120 |
| DL-Glyceraldehyde 3-phosphate | 591-59-3 | C3H7O6P | Negative | 169 | 79 | -110 | -45 |
| Urate | 69-93-2 | C5H4N4O3 | Negative | 167 | 124 | -190 | -20 |
| Pyruvate | 127-17-3 | C3H4O3 | Negative | 87 | 32 | -40 | -13 |
| α-Keto-glutarate | 328-50-7 | C5H6O5 | Negative | 145 | 101 | -100 | -18 |
| Thymine | 65-71-4 | C5H6N2O2 | Negative | 125 | 42 | -100 | -32 |
| Fumarate | 110-17-8 | C4H4O4 | Negative | 115 | 71 | -40 | -11 |
| L-Gulono-1,4-lactone | 1128-23-0 | C6H10O6 | Negative | 177 | 89 | -120 | -17 |
| Xanthine | 69-89-6 | C5H4N4O2 | Negative | 151.1 | 108 | -140 | -22 |
| Biotin | 58-85-5 | C10H16N2O3S | Negative | 243.2 | 200 | -120 | -21 |
| Benzoate | 65-85-0 | C7H6O2 | Negative | 120.9 | 77.1 | -60 | -10 |
| Mevalonate | 150-97-0 | C6H12O4 | Negative | 147.1 | 59 | -110 | -37 |
| Orotate | 65-86-1 | C5H4N2O4 | Negative | 155 | 111 | -50 | -17 |
| N-acetyl-L-glutamate | 1188-37-0 | C7H11NO5 | Negative | 188 | 102 | -40 | -24 |
| 2-Oxoglutarate | 328-50-7 | C5H6O5 | Negative | 145.1 | 101 | -90 | -17 |
| L-(-)-Malic acid | 97-67-6 | C4H6O5 | Negative | 133 | 115 | -70 | -16 |
| Phenylacetylglycine | 500-98-1 | C10H11NO3 | Negative | 192 | 74 | -60 | -10 |
| L-Cysteic acid | 23537-25-9 | C3H9NO6S | Negative | 168.1 | 81 | -100 | -56 |
| OH-Phenylpyruvate | 156-39-8 | C9H8O4 | Negative | 179.2 | 107 | -45 | -12 |
| D-glucose 6-phosphate | 56-73-5 | C6H13O9P | Negative | 258.8 | 96.8 | -60 | -10 |
| 3',5'-Cyclic AMP | 60-92-4 | C10H12N5O6P | Negative | 328 | 134 | -60 | -30 |
| N-Acetylneuraminate | 131-48-6 | C11H19NO9 | Negative | 308.2 | 170 | -70 | -19 |
| NADH | 58-68-4 | C21H29N7O14P2 | Negative | 663.2 | 79 | -120 | -128 |
| Folate | 59-30-3 | C19H19N7O6 | Negative | 440.1 | 311 | -120 | -33 |
| Sucrose | 57-50-1 | C12H22O11 | Negative | 341 | 89 | -120 | -25 |
| D-Fructose 6-phosphate | 643-13-0 | C6H13O9P | Negative | 259 | 79 | -80 | -60 |
| L-Lactic Acid | 79-33-4 | C3H6O3 | Negative | 89 | 43 | -100 | -25 |
| Xanthosine | 146-80-5 | C10H12N4O6 | Negative | 283.1 | 108 | -100 | -50 |
| Lipoate | 1200-22-2 | C8H14O2S2 | Negative | 205 | 170.8 | -60 | -10 |
| UDP-glucose | 133-89-1 | C15H24N2O17P2 | Negative | 565 | 79 | -150 | -120 |
| D-Glucose 1-phosphate | 59-56-3 | C6H13O9P | Negative | 259 | 79 | -120 | -120 |
| D-Ribose | 50-69-1 | C5H10O5 | Negative | 149 | 89 | -110 | -10 |
| Maleate | 110-16-7 | C4H4O4 | Negative | 115 | 71 | -100 | -26 |
| N-Acetyl-Asp-Glu | 3106-85-2 | C11H16N2O8 | Negative | 303.2 | 96 | -80 | -54 |
| 1-Methyluric acid | 708-79-2 | C6H6N4O3 | Negative | 181 | 138 | -80 | -20 |
| IMP | 352195-40-5 | C10H13N4O8P | Negative | 347 | 79 | -120 | -80 |
| D-Erythrose 4-phosphate | 585-18-2 | C4H9O7P | Negative | 199 | 97 | -55 | -15 |
| Dihydrofolate | 4033-27-6 | C19H21N7O6 | Negative | 442.1 | 176 | -110 | -35 |
| ESTRIOL E3 | 50-27-1 | C18 H24 O3 | Negative | 287 | 143 | -190 | -70 |
| 17-hydroxypregnenolone | 387-79-1 | C21 H32 O3 | Negative | 331 | 287 | -150 | -26 |
| ESTRONE E1 | 53-16-7 | C18 H22 O2 | Negative | 269 | 145 | -180 | -48 |
| Pregnenolone | 145-13-1 | C21 H32 O2 | Negative | 315.2 | 282.2 | -230 | -30 |
| UMP | 3387-36-8 | C9H13N2O9P | Negative | 323 | 79 | -100 | -80 |
| FA 12:0 | 143-07-7 | C12H24O2 | Negative | 199.2 | 199.2 | -100 | -10 |
| FA 14:0 | 544-63-8 | C14H28O2 | Negative | 227.2 | 227.2 | -100 | -10 |
| FA 16:4 | 29259-52-7 | C16H24O2 | Negative | 247.2 | 247.2 | -100 | -10 |
| FA 16:1 | 373-49-9 | C16H30O2 | Negative | 253.2 | 253.2 | -100 | -10 |
| FA 16:0 | 57-10-3 | C16H32O2 | Negative | 255.2 | 255.2 | -100 | -30 |
| FA 17:1 | 29743-97-3 | C17H32O2 | Negative | 267.2 | 267.2 | -100 | -10 |
| FA 17:0 | 506-12-7 | C17H34O2 | Negative | 269.2 | 269.2 | -100 | -10 |
| FA 18:4 | 20290-75-9 | C18H28O2 | Negative | 275.2 | 275.2 | -100 | -10 |
| FA 18:3-α | 463-40-1 | C18H30O2 | Negative | 277.2 | 277.2 | -100 | -30 |
| FA 18:3-γ | 506-26-3 | C18H30O2 | Negative | 277.2 | 277.2 | -100 | -10 |
| FA 18:2 | 60-33-3 | C18H32O2 | Negative | 279.2 | 279.2 | -100 | -30 |
| FA 18:1 | 112-80-1 | C18H34O2 | Negative | 281.2 | 281.2 | -100 | -30 |
| FA 18:0 | 57-11-4 | C18H36O2 | Negative | 283.2 | 283.2 | -100 | -30 |
| FA 20:5 | 10417-94-4 | C20H30O2 | Negative | 301.2 | 301.2 | -100 | -10 |
| FA 20:4 | 506-32-1 | C20H32O2 | Negative | 303.2 | 303.2 | -100 | -30 |
| FA 20:3 | 1783-84-2 | C20H34O2 | Negative | 305.2 | 305.2 | -100 | -10 |
| FA 20:2 | 135498-07-6 | C20H36O2 | Negative | 307.2 | 307.2 | -100 | -10 |
| FA 20:1 | 62322-84-3 | C20H38O2 | Negative | 309.2 | 309.2 | -100 | -10 |
| FA 20:0 | 506-30-9 | C20H40O2 | Negative | 311.2 | 311.2 | -100 | -10 |
| FA 22:6 | 6217-54-5 | C22H32O2 | Negative | 327.2 | 327.2 | -100 | -30 |
| FA 22:5- n3 | 24880-45-3 | C22H34O2 | Negative | 329.2 | 329.2 | -100 | -10 |
| FA 22:5- n6 | 25182-74-5 | C22H34O2 | Negative | 329.2 | 329.2 | -100 | -10 |
| FA 22:4 | - | C22H36O2 | Negative | 331.2 | 331.2 | -100 | -10 |
| FA 22:3-iso1 | 28845-86-5 | C22H38O2 | Negative | 333.2 | 333.2 | -100 | -10 |
| FA 22:2 | 17735-98-7 | C22H40O2 | Negative | 335.2 | 335.2 | -100 | -10 |
| FA 22:1 | 112-86-7 | C22H42O2 | Negative | 337.2 | 337.2 | -100 | -10 |
| FA 22:0 | 112-85-6 | C22H44O2 | Negative | 339.2 | 339.2 | -100 | -10 |
| Dehydrolithocholic acid | 1553-56-6 | C24H38O3 | Negative | 373.3 | 373.3 | -150 | -20 |
| Lithocholic acid | 434-13-9 | C24H40O3 | Negative | 375.3 | 375.3 | -150 | -15 |
| Alloisolithocholic acid | 2276-93-9 | C24H40O3 | Negative | 375.3 | 375.3 | -150 | -20 |
| Isolithocholic acid | 1534-35-6 | C24H40O3 | Negative | 375.3 | 375.3 | -150 | -20 |
| Nordeoxycholic acid | 53608-86-9 | C23H38O4 | Negative | 377.3 | 377.3 | -150 | -20 |
| 5α-cholanic acid-3,6-dione | 6929-22-2 | C24 H36 O4 | Negative | 387.3 | 387.3 | -150 | -15 |
| 3,7-Diketocholanic acid | 859-97-2 | C24 H36 O4 | Negative | 387.3 | 387.3 | -150 | -20 |
| Apocholic acid | 641-81-6 | C24H38O4 | Negative | 389.3 | 389.3 | -150 | -20 |
| 5α-cholanic acid-3α-ol-6-one | 10573-17-8 | C24 H38 O4 | Negative | 389.3 | 389.3 | -150 | -20 |
| 7-Ketolithocholic acid | 4651-67-6 | C24 H38 O4 | Negative | 389.3 | 389.3 | -150 | -20 |
| 12-Ketolithocholic acid | 5130-29-0 | C24 H38 O4 | Negative | 389.3 | 389.3 | -150 | -20 |
| Isodeoxycholic acid | 566-17-6 | C24H40O4 | Negative | 391.3 | 391.3 | -150 | -15 |
| Deoxycholic acid | 83-44-3 | C24H40O4 | Negative | 391.3 | 391.3 | -150 | -20 |
| Ursodeoxycholic acid | 128-13-2 | C24H40O4 | Negative | 391.3 | 391.3 | -150 | -20 |
| Hyodeoxycholic acid | 83-49-8 | C24H40O4 | Negative | 391.3 | 391.3 | -150 | -20 |
| Dehydrocholic acid | 81-23-2 | C24H34O5 | Negative | 401.3 | 401.3 | -150 | -20 |
| Dioxolithocholic acid | 517-33-9 | C24H36O5 | Negative | 403.3 | 403.3 | -150 | -20 |
| 6,7-Diketolithocholic acid | - | C24H36O5 | Negative | 403.3 | 403.3 | -150 | -20 |
| 12-Ketochenodeoxycholic acid | 2458-08-4 | C24H38O5 | Negative | 405.3 | 405.3 | -260 | -20 |
| Cholic acid | 81-25-4 | C24H40O5 | Negative | 407.3 | 407.3 | -150 | -20 |
| Glycoursocholanic acid | - | C26H43NO3 | Negative | 416.3 | 416.3 | -150 | -20 |
| Glycodeoxycholic acid | 360-65-6 | C26H43NO5 | Negative | 448.3 | 448.3 | -150 | -15 |
| Glycoursodeoxycholic acid | 64480-66-6 | C26H43NO5 | Negative | 448.3 | 448.3 | -150 | -15 |
| Glycochenodeoxycholic acid | 640-79-9 | C26H43NO5 | Negative | 448.3 | 448.3 | -150 | -20 |
| Glycohyodeoxycholic acid | 13042-33-6 | C26H43NO5 | Negative | 448.3 | 448.3 | -150 | -20 |
| Glycodehydrocholic acid | - | C26H37NO6 | Negative | 458.3 | 458.3 | -150 | -15 |
| Glycocholic acid | 475-31-0 | C26H43NO6 | Negative | 464.3 | 464.3 | -150 | -15 |
| Taurolithocholic acid | - | C26H45NO5S | Negative | 482.5 | 80 | -150 | -110 |
| Tauroursodeoxycholic acid/Taurohyodeoxycholic acid | 14605-22-2 | C26H45NO6S | Negative | 498.3 | 80 | -150 | -120 |
| Taurodeoxycholic acid | 516-50-7 | C26H45NO6S | Negative | 498.3 | 80 | -150 | -120 |
| Taurochenodeoxycholic acid | 516-35-8 | C26H45NO6S | Negative | 498.5 | 80 | -150 | -120 |
| Taurodehydrocholic acid | 517-37-3 | C26H39NO7S | Negative | 508.5 | 80 | -150 | -120 |
| Taurocholic acid | 81-24-3 | C26H45NO7S | Negative | 514.5 | 80 | -150 | -140 |
