# Supplementary material for: Dose- and time-dependent manners of moxifloxacin induced liver injury by targeted metabolomics study
Source: Front Pharmacol. 2022 Sep 16;13:994821. doi: 10.3389/fphar.2022.994821 (PMC9525095; doi:10.3389/fphar.2022.994821)
Supplement: Supplementary file 1 [file DataSheet1.zip › supplementary materials/Table S2.docx]

**Table S1.** Significantly changed metabolites between control and 7-days HD group, with adjust P value less than 0.05 and FCs larger than 2.

| **Metabolites** | **HMDB ID** | **FC (Contro/HD)** | **log2(FC)** | **P value** |
| --- | --- | --- | --- | --- |
| DL 14:1-iso2 |  | 0.090029 | -3.4735 | 5.50E-08 |
| DL 10:1-iso2 |  | 0.11928 | -3.0675 | 5.50E-08 |
| DL 16:1-iso1 |  | 0.07462 | -3.7443 | 8.02E-08 |
| Fumarycarnitine-iso3 | HMDB0013134 | 0.13253 | -2.9156 | 2.56E-07 |
| DL 16:2 |  | 0.10177 | -3.2967 | 3.03E-07 |
| IMP | HMDB0000175 | 0.0034535 | -8.1777 | 6.14E-07 |
| DL 14:1-iso1 |  | 0.093424 | -3.4201 | 6.14E-07 |
| Octanoyl-L-carnitine | HMDB0000791 | 0.1356 | -2.8826 | 6.14E-07 |
| Nordeoxycholic acid | HMDB0304947 | 2.7794 | 1.4748 | 6.14E-07 |
| DL 10:0 | HMDB0000651 | 0.1304 | -2.939 | 6.25E-07 |
| DL 16:0 | HMDB0000222 | 0.16817 | -2.572 | 6.67E-07 |
| DL 14:2 |  | 0.15309 | -2.7075 | 8.47E-07 |
| DL 14:0 | HMDB0005066 | 0.12717 | -2.9752 | 1.28E-06 |
| DL 12:0 | HMDB0002250 | 0.16513 | -2.5983 | 1.32E-06 |
| DL 10:1-iso1 |  | 0.12266 | -3.0272 | 2.08E-06 |
| DL 18:1 | HMDB0005065 | 0.12617 | -2.9865 | 2.08E-06 |
| DL 18:2 | HMDB0006469 | 0.14747 | -2.7615 | 2.08E-06 |
| DL 13:0-iso1 |  | 0.1184 | -3.0782 | 2.96E-06 |
| O-Acetyl-L-carnitine | HMDB0000201 | 0.27245 | -1.8759 | 2.96E-06 |
| DL 6:1-iso2 | HMDB0013161 | 0.18761 | -2.4142 | 3.71E-06 |
| DL 18:3-iso2 | HMDB0006319 | 0.22152 | -2.1745 | 4.16E-06 |
| FA 20:1-iso1 | HMDB0034296 | 0.3382 | -1.564 | 4.68E-06 |
| FA 18:0 | HMDB0000827 | 0.49594 | -1.0118 | 5.55E-06 |
| DL 8:1-iso2 |  | 0.13804 | -2.8568 | 5.75E-06 |
| Spermidine | HMDB0001257 | 0.12872 | -2.9577 | 5.77E-06 |
| DL 18:0 | HMDB0000848 | 0.25806 | -1.9542 | 6.24E-06 |
| FA 20:3-iso3 |  | 0.19806 | -2.336 | 1.31E-05 |
| GMP | HMDB0001397 | 0.013182 | -6.2453 | 1.44E-05 |
| Hippurate | HMDB0000714 | 23.935 | 4.5811 | 1.44E-05 |
| DL 8:1-iso3 |  | 0.1392 | -2.8448 | 1.44E-05 |
| DL 18:3-iso1 |  | 0.16625 | -2.5886 | 1.44E-05 |
| FA 16:0 | HMDB0000220 | 0.4375 | -1.1926 | 1.44E-05 |
| FA 14:0 | HMDB0000806 | 0.39511 | -1.3397 | 1.54E-05 |
| DL 13:0-iso2 |  | 0.23559 | -2.0856 | 1.79E-05 |
| AMP | HMDB0000045 | 0.001241 | -9.6543 | 2.66E-05 |
| Glycochenodeoxycholic acid | HMDB0000637 | 0.054356 | -4.2014 | 2.82E-05 |
| FA 18:1 | HMDB0000207 | 0.3241 | -1.6255 | 3.03E-05 |
| Fumarycarnitine-iso1 |  | 0.28758 | -1.798 | 3.18E-05 |
| Homocystine | HMDB0000676 | 3.4035 | 1.767 | 4.00E-05 |
| FA 17:0 | HMDB0002259 | 0.42716 | -1.2272 | 4.00E-05 |
| Spermine | HMDB0001256 | 0.075845 | -3.7208 | 4.70E-05 |
| Folate | HMDB0000121 | 11.112 | 3.4741 | 5.29E-05 |
| DL 10:3-iso1 |  | 0.19702 | -2.3436 | 7.13E-05 |
| FA 22:5- n3 | HMDB0006528 | 0.41296 | -1.2759 | 0.00010207 |
| Hyodeoxycholic acid | HMDB0000733 | 5.9259 | 2.567 | 0.00010637 |
| Dihydrofolate | HMDB0001056 | 4.7232 | 2.2398 | 0.00010637 |
| FA 17:1 | HMDB0060038 | 0.28837 | -1.794 | 0.00010637 |
| FA 22:6 | HMDB0002183 | 0.30175 | -1.7286 | 0.00010637 |
| Cytidine | HMDB0000089 | 0.41929 | -1.254 | 0.00010637 |
| D-Ribose | HMDB0000283 | 2.8792 | 1.5257 | 0.00012315 |
| FA 18:2 | HMDB0000673 | 0.39475 | -1.341 | 0.00012315 |
| FA 22:4 | HMDB0002226 | 0.40055 | -1.3199 | 0.0001312 |
| DL 8:1-iso1 |  | 0.19451 | -2.3621 | 0.00014268 |
| DL 10:3-iso2 |  | 0.26199 | -1.9324 | 0.00019123 |
| FA 16:1 | HMDB0003229 | 0.19581 | -2.3525 | 0.00019644 |
| Creatinine | HMDB0000562 | 0.35658 | -1.4877 | 0.00019644 |
| DL 8:0 | HMDB0000791 | 0.25782 | -1.9556 | 0.00020365 |
| Guanosine | HMDB0000133 | 3.2301 | 1.6916 | 0.00026913 |
| Cytosine | HMDB0000630 | 3.8984 | 1.9629 | 0.00051336 |
| FA 20:4 | HMDB0001043 | 0.45896 | -1.1236 | 0.00062442 |
| Apocholic acid |  | 5.5188 | 2.4644 | 0.00072344 |
| FA 20:5 | HMDB0001999 | 0.42946 | -1.2194 | 0.0007772 |
| Pyruvate | HMDB0000243 | 0.46327 | -1.1101 | 0.00080571 |
| FA 18:3-n6 | HMDB0003073 | 0.4546 | -1.1373 | 0.0008208 |
| Valerylcarnitine-iso1 | HMDB0013128 | 0.40919 | -1.2892 | 0.00086921 |
| FA 18:3-n3 | HMDB0001388 | 0.31313 | -1.6752 | 0.00091351 |
| Glycocholic acid | HMDB0000138 | 0.089295 | -3.4853 | 0.0010993 |
| FA 18:4 | HMDB0006547 | 0.34809 | -1.5225 | 0.0011433 |
| 3,5-Cyclic AMP | HMDB0000058 | 0.040272 | -4.6341 | 0.0011572 |
| Ascorbic acid | HMDB0000044 | 46.802 | 5.5485 | 0.0013369 |
| L-Cystine | HMDB0000192 | 2.0922 | 1.065 | 0.0013369 |
| 12-Ketolithocholic acid | HMDB0000328 | 2.9911 | 1.5807 | 0.0014709 |
| FA 22:5- n6 | HMDB0001976 | 0.34831 | -1.5215 | 0.0014709 |
| Homocysteine | HMDB0000742 | 0.43896 | -1.1878 | 0.001733 |
| Valerylcarnitine-iso2 | HMDB0013128 | 0.40199 | -1.3148 | 0.0018187 |
| FA 20:3-iso2 | HMDB0002925 | 0.49817 | -1.0053 | 0.0020018 |
| FA 22:3-iso2 | HMDB0002823 | 0.49325 | -1.0196 | 0.002475 |
| OH-Phenylpyruvate | HMDB0000707 | 4.2984 | 2.1038 | 0.0029765 |
| Fumarycarnitine-iso2 |  | 0.37175 | -1.4276 | 0.0045035 |
| 1,3-Diaminopropane | HMDB0000002 | 0.18454 | -2.438 | 0.0049054 |
| Reduced glutathione | HMDB0000125 | 0.26125 | -1.9365 | 0.0066614 |
| Propionylcarnitine | HMDB0000824 | 0.46847 | -1.094 | 0.0073837 |
| Ornithine | HMDB0000214 | 2.2583 | 1.1752 | 0.01001 |
| Cholic acid | HMDB0000619 | 2.4729 | 1.3062 | 0.010664 |
| Glycoursodeoxycholic acid | HMDB0000708 | 0.18165 | -2.4608 | 0.011965 |
| dehydroepiandrosterone | HMDB0000077 | 0.073237 | -3.7713 | 0.013795 |
| 12-Ketochenodeoxycholic acid | HMDB0000400 | 0.35593 | -1.4903 | 0.015936 |
| D-Gluconic acid | HMDB0000625 | 2.2026 | 1.1392 | 0.016274 |
| N-Acetylneuraminate | HMDB0000230 | 2.34 | 1.2265 | 0.017718 |
| D-Glucosamine 6-phosphate | HMDB0250754 | 0.46201 | -1.114 | 0.018374 |
| Cysteine | HMDB0000574 | 0.25781 | -1.9556 | 0.020857 |
| N-Acetyl-D-glucosamine | HMDB0000215 | 3.4911 | 1.8037 | 0.020857 |
| cGMP | HMDB0001314 | 0.025233 | -5.3086 | 0.022682 |
| Valerylcarnitine-iso3 |  | 0.2939 | -1.7666 | 0.022876 |
| 2-Oxoglutarate | HMDB0000208 | 0.49633 | -1.0106 | 0.023796 |
| L-Carnosine | HMDB0000033 | 4.0083 | 2.003 | 0.028464 |
| Deoxycholic acid | HMDB0000626 | 2.8278 | 1.4997 | 0.035244 |
| L-(-)-Malic acid | HMDB0000156 | 0.48513 | -1.0436 | 0.035396 |
| Oxidized glutathione | HMDB0003337 | 0.48902 | -1.032 | 0.043987 |
| Taurochenodeoxycholic acid | HMDB0000951 | 3.1464 | 1.6537 | 0.046196 |
| Trimethylamine-N-oxide | HMDB0000925 | 2.4794 | 1.31 | 0.048554 |
