# Supplementary material for: Dose- and time-dependent manners of moxifloxacin induced liver injury by targeted metabolomics study
Source: Front Pharmacol. 2022 Sep 16;13:994821. doi: 10.3389/fphar.2022.994821 (PMC9525095; doi:10.3389/fphar.2022.994821)
Supplement: Supplementary file 1 [file DataSheet1.zip › supplementary materials/Table S3.docx]

**Table S2.** Pearson’s correlation analysis of dehydroepiandrosterone with fatty acyl carnitines and fatty acids.

| **Metabolites** | **Correlation coefficient** |
| --- | --- |
| Dehydroepiandrosterone | 1.000 |
| Propionylcarnitine | 0.577** |
| Butenylcarnitine-iso1 | 0.594** |
| Valerylcarnitine-iso1 | 0.757** |
| Valerylcarnitine-iso2 | 0.671** |
| Valerylcarnitine-iso3 | 0.574** |
| Fumarycarnitine-iso1 | 0.739** |
| Fumarycarnitine-iso2 | 0.667** |
| Fumarycarnitine-iso3 | 0.932** |
| DL 8:0 | 0.591** |
| DL 5:1 | 0.674** |
| DL 6:1-iso1 | 0.553** |
| DL 6:1-iso2 | 0.899** |
| DL 8:1-iso1 | 0.508** |
| DL 8:1-iso2 | 0.586** |
| DL 8:1-iso3 | 0.947** |
| DL 10:3-iso1 | 0.668** |
| DL 10:3-iso2 | 0.642** |
| DL 10:1-iso1 | 0.938** |
| DL 10:1-iso2 | 0.950** |
| DL 10:0 | 0.964** |
| DL 12:0 | 0.923** |
| DL 13:0-iso1 | 0.752** |
| DL 13:0-iso2 | 0.742** |
| DL 14:2 | 0.883** |
| DL 14:1-iso1 | 0.887** |
| DL 14:1-iso2 | 0.930** |
| DL 14:0 | 0.874** |
| DL 16:2 | 0.849** |
| DL 16:1-iso1 | 0.886** |
| DL 16:0 | 0.859** |
| DL 18:0 | 0.762** |
| DL 18:1 | 0.905** |
| DL 18:2 | 0.881** |
| DL 18:3-iso1 | 0.786** |
| DL 18:3-iso2 | 0.690** |
| O-Acetyl-L-carnitine | 0.908** |
| Carnitine | 0.640** |
| Octanoyl-L-carnitine | 0.986** |
| FA 12:0 | 0.514** |
| FA 14:0 | 0.796** |
| FA 16:1 | 0.687** |
| FA 16:0 | 0.759** |
| FA 17:1 | 0.744** |
| FA 17:0 | 0.620** |
| FA 18:4 | 0.310** |
| FA 18:3-n3 | 0.523** |
| FA 18:3-n6 | 0.232* |
| FA 18:2 | 0.604** |
| FA 18:1 | 0.779** |
| FA 18:0 | 0.772** |
| FA 20:5 | 0.247* |
| FA 20:4 | 0.570** |
| FA 20:3-iso2 | 0.456** |
| FA 20:3-iso3 | 0.620** |
| FA 20:2 | 0.429** |
| FA 20:1-iso1 | 0.672** |
| FA 22:6 | 0.551** |
| FA 22:5- n3 | 0.277** |
| FA 22:5- n6 | 0.317** |
| FA 22:4 | 0.360** |
| FA 22:3-iso1 | 0.248* |
| FA 22:3-iso2 | 0.335** |
| FA 22:2 | 0.244* |

Only the correlations with P<0.05 were listed in the table. *, P<0.05; **, P<0.01.
